# Definition of erythroid cell‐positive blood transcriptome phenotypes associated with severe respiratory syncytial virus infection

**DOI:** 10.1002/ctm2.244

**Published:** 2020-12-12

**Authors:** Darawan Rinchai, Matthew C. Altman, Oceane Konza, Signe Hässler, Federica Martina, Mohammed Toufiq, Mathieu Garand, Basirudeen Syed Ahamed Kabeer, Karolina Palucka, Asuncion Mejias, Octavio Ramilo, Davide Bedognetti, Encarnita Mariotti‐Ferrandiz, David Klatzmann, Damien Chaussabel

**Affiliations:** ^1^ Sidra Medicine Doha Qatar; ^2^ Benaroya Research Institute Seattle Washington; ^3^ University of Washington Seattle Washington; ^4^ Biotherapy (CIC‐BTi) and Inflammation‐Immunopathology‐Biotherapy Department (i2B) AP‐HP, Hôpital Pitié‐Salpêtrière Paris France; ^5^ Immunology‐Immunopathology‐Immunotherapy (i3) Sorbonne Université INSERM Paris France; ^6^ Jackson Laboratory for Genomic Medicine Farmington Connecticut; ^7^ Division of Infectious Diseases Nationwide Children's Hospital Columbus Ohio; ^8^ Department of Internal Medicine and Medical Specialties University of Genoa Genoa Italy

## Abstract

Biomarkers to assess the risk of developing severe respiratory syncytial virus (RSV) infection are needed. We conducted a meta‐analysis of 490 unique profiles from six public RSV blood transcriptome datasets. A repertoire of 382 well‐characterized transcriptional modules was used to define dominant host responses to RSV infection. The consolidated RSV cohort was stratified according to four traits: “interferon response” (IFN), “neutrophil‐driven inflammation” (Infl), “cell cycle” (CC), and “erythrocytes” (Ery). We identified eight prevalent blood transcriptome phenotypes, of which three Ery+ phenotypes comprised higher proportions of patients requiring intensive care. This finding confirms on a larger scale data from one of our earlier reports describing an association between an erythrocyte signature and RSV disease severity. Further contextual interpretation made it possible to associate this signature with immunosuppressive states (late stage cancer, pharmacological immunosuppression), and with a population of fetal glycophorin A+ erythroid precursors. Furthermore, we posit that this erythrocyte cell signature may be linked to a population of immunosuppressive erythroid cells previously described in the literature, and that overabundance of this cell population in RSV patients may underlie progression to severe disease. These findings outline potential priority areas for biomarker development and investigations into the immune biology of RSV infection. The approach that we developed and employed here should also permit to delineate prevalent blood transcriptome phenotypes in other settings.

## Graphical abstract

Immune determinants of respiratory syncytial virus (RSV) disease severity remain ill‐defined. We conducted a meta‐analysis of six public RSV blood transcriptome datasets. Severe patients molecular phenotypes presented traits associated with immunosuppressive states and extramedullary erythropoiesis signatures.

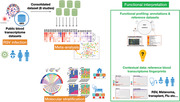

## 1 INTRODUCTION

Respiratory syncytial virus (RSV) infection is the leading cause of hospitalization and the second cause of infant mortality worldwide.^1^ There are well‐characterized populations at risk for severe disease, but most infants who develop a severe RSV infection have no underlying health conditions.^2^, ^3^ The mechanisms underlying RSV morbidity are poorly understood, but studies suggest that immature or underdeveloped lungs and/or a dysregulated immune response might have a role.^4^

Several groups of researchers, including us, have undertaken blood transcriptome profiling studies of patients with RSV infection.^5^, ^6^, ^7^, ^8^, ^9^, ^10^, ^11^, ^12^, ^13^ This approach involves measuring the abundance of blood leukocyte transcripts on a genome‐wide scale.^14^, ^15^ Whole blood comprises a heterogeneous mix of leukocyte populations; thus, changes in transcript abundance might be attributable to either gene expression regulation or relative changes in cell abundance. Regardless, blood transcriptome profiling remains one of the most straightforward approaches to implement in clinical settings and on a large scale.^15^ Among the RSV blood transcriptome studies, several aimed to identify factors associated with severe disease. For example, we reported an increase in abundance of neutrophil, inflammation, and erythrocyte genes in severe pediatric cases.^7^ Brand et al pinpointed that an increase in abundance of transcripts coding for olfactomedin 4, a factor involved in inflammatory responses, is strongly associated with disease severity.^10^ More recently, Do et al linked RSV disease severity with follicular T‐helper‐cell development and BCL6‐dependant inflammation.^9^

Building consensus around biomarker signatures and finding a path to clinical utility may involve performing meta‐analyses that regroup datasets derived from multiple independent studies.^16^ Work that permitted the development of novel diagnostic products for sepsis provides a good example.^17^, ^18^ An obvious benefit of consolidating data from multiple studies is that it permits to achieve larger sample sizes. Arguably, heterogeneity of the patient populations and clinical settings could also help to improve the robustness of the resulting biomarker signatures.^16^ Challenges include the presence of important technical variability between studies, such as in the sampling methods, the profiling platform used, or data preprocessing. Another potential limitation is the varying depth and lack of harmonization of sample or subject information available from one study to another.

Such meta‐analyses tend to focus on the identification of consensus biomarker signatures where the deliverable is a set of differentially expressed genes or predictors of a given clinical outcome. In the present work, we endeavored to identify discrete molecular traits (eg, “interferon = IFN,” “inflammation = Inf,” “erythrocytes” = Ery”) underlying interindividual differences among patients with RSV infection. We used such traits to define blood transcriptome phenotypes and to stratify patient cohorts on the basis of their individual status of each trait: increased, decreased, or unchanged versus the uninfected comparators (eg, of a given phenotype being IFN+ Infl0 Ery−). The next key step was to assess the clinical relevance of such a classification, for instance in terms of differences in the degrees of RSV disease severity. We finally endeavored to investigate biological basis of the interindividual variation being measured, especially for the traits showing the highest degree of association with severe presentations.

A fixed repertoire of transcriptional modules formed the basis for this work. This repertoire consists of a collection of coexpressed gene sets. Coexpression was determined in a collection of reference datasets encompassing 16 distinct immunological states^19^ (see Section 4). This 382‐module repertoire is “fixed” in the sense that it serves as a reusable framework to analyze and interpret transcriptome data. As such, transcriptional modules are not re‐formed every time a new dataset is analyzed. Reducing the number of variables by using said modules permits the selection of discrete molecular traits. These traits can in turn be used for patient phenotyping and cohort stratification. The fact that this module repertoire is fixed and reused across studies also justifies investing more time for its functional characterization than would be customary. Extensive annotations frameworks such as the one we have developed can in turn prove especially valuable when attempting to discern the biological significance of patient blood transcriptome phenotypes.

In summary, we present here a meta‐analysis of six RSV blood transcriptome datasets that include 490 unique subject profiles. Specifically, we aimed to: (a) measure interindividual variability and molecularly stratify RSV patients; (b) identify the associations between patient molecular phenotypes, clinical parameters, and outcomes; and (c) identify and interpret the immunobiological processes associated with each molecular phenotype.

## 2 RESULTS

### 2.1 A collection of RSV blood transcriptome datasets can be assembled from earlier submissions to public repositories

Several researchers investigating the host responses to RSV infection have made their blood transcriptome datasets public. We consolidated the datasets contributed by six independent studies,^5^, ^6^, ^7^, ^20^, ^21^, ^22^ and performed meta‐analyses to delineate distinct blood transcriptome phenotypes among RSV subjects. A criterion for including studies in this meta‐analysis was the availability of uninfected controls: this point is important because control groups serve as a common denominator between studies and provide the basis for data normalization. Thus, public datasets for which such controls were unavailable could not be included in this meta‐analysis.

As each study had different goals and designs, it was first important to identify the key differences so that they are accounted for when interpreting the meta‐analysis results. Information about the six studies is summarized in Table 1. The most notable outlier in this collection was the dataset from Liu et al (GSE73072^21^), as it consisted of samples from adult subjects collected before and after experimental exposure to RSV. We decided to retain this dataset as it met our initial screening criterion (availability of uninfected control samples), and would help us to maximize the sample size, which was our priority. Importantly, downstream analyses revealed that the RSV disease signature in this adult population was largely consistent with that observed in pediatric cohorts. This observation carries notable implications that we elaborate on later in Section 3. All other studies comprised pediatric subjects with community‐acquired RSV infection and a separate group of uninfected controls. Among the latter, the study by Mejias et al addressed the question of disease severity most directly (GSE38900^7^), while the work of de Steenhuijsen Piters et al (GSE77087^6^) examined the effects of microbiome composition on the disease course and blood transcriptome signatures. Rodriguez‐Fernandez et al examined the influence of RSV genotypes on blood transcriptional signatures (GSE103842^5^), while McDonald et al focused on identifying pathways involved in disease pathogenesis (GSE80179^22^). In the study by Herberg et al (GSE42026^20^), the RSV dataset was used as a comparator in a study focusing on responses to H1N1 influenza. The latter two studies were conducted in Europe, while all others were conducted in the United States of America. Finally, in terms of technical variables, samples from the adult exposure study were run using Affymetrix GeneChips, while the others were run on Illumina BeadArrays. The sample types were otherwise homogenous across all studies and consisted of RNA‐stabilized whole blood. The studies used one of two popular commercial sample collection tubes for this type of application: PAXgene blood RNA tubes (three studies) or Tempus tubes (three studies).

**TABLE 1:** Description of the public respiratory syncytial virus (RSV) blood transcriptome datasets

**GEO ID**	**Reference**	**Title**	**N Subjects**	**Age demographics**	**Sample type**	**Platform**	Cluster# (Figure >1A)
GSE42026	Herberg et al/23901082/^20^	Transcriptomic profiling in childhood H1N1/09 influenza reveals reduced expression of protein synthesis genes	22 RSV and 33 controls	Pediatric	Whole blood (PAXgene)	Illumina Human HT‐12 V3.0	1
GSE38900	Mejias et al/24265599/^7^	Genome‐wide analysis of whole blood transcriptional response to RSV, influenza, and rhinovirus (LRTI) in children	107 RSV and 31 controls	Pediatric	Whole blood (Tempus)	Illumina Human HT‐12 V3.0	1
GSE80179	McDonald et al/27822537/^22^	A simple screening approach to prioritize genes for functional analysis identifies a role for IRF7 in the control of RSV disease.	27 RSV and 52 controls	Pediatric	Whole blood (PAXgene)	Illumina Human HT‐12 V4.0	1
GSE73072	Liu et al/26801061/^21^	Host gene expression signatures of H1N1, H3N2, HRV, RSV virus infection in adults	20 subjects 2 time points	Adult	Whole blood (PAXgene)	Affymetrix GeneChip Human Genome U133A 2.0 Array	2
GSE77087	de Steenhuijsen Piters et al/27135599/^6^	Nasopharyngeal microbiota, host transcriptome, and disease severity in children with RSV infection	81 RSV and 23 controls	Pediatric	Whole blood (Tempus)	Illumina Human HT‐12 V4.0	2
GSE103842	Rodriguez‐Fernandez et al/29045741/^5^	RSV genotypes and disease severity in young children hospitalized with bronchiolitis	62 RSV and 12 controls	Pediatric	Whole blood (Tempus)	Illumina Human HT‐12 V4.0	2

Altogether, the consolidated dataset collection that was constituted for this meta‐analysis encompassed 490 profiles, of which 319 were from subjects with RSV infection. We hypothesized that this expanded sample size should permit us to define blood transcriptome phenotypes, and stratify patient cohorts more effectively than each individual study could.

### 2.2 Comparing the changes in transcript abundance across module aggregates identifies a consensus RSV signature

Meta‐analyses are compounded by a high degree of technical variation existing between independent studies. To at least partly address such challenges, we used a fixed repertoire of transcriptional modules^19^ as a framework for data analysis and interpretation. In brief, this repertoire comprises 382 modules, each formed by a set of genes grouped together based on patterns of coexpression across a reference collection of 16 blood transcriptome datasets. This collection was comprised of 985 unique transcriptome profiles and spanned 16 different immunologically relevant pathological or physiological states (see Methods section). Dimensions were further reduced by organizing, in turn, the modules into 38 “aggregates” (designated A1‐A38). This grouping was based on similarities in patterns of transcript abundance, determined this time at the module level and across the 16 reference datasets. Each aggregate comprised between 1 and 42 modules (27 of the 38 aggregates comprised two modules or more).

**FIGURE 1:**
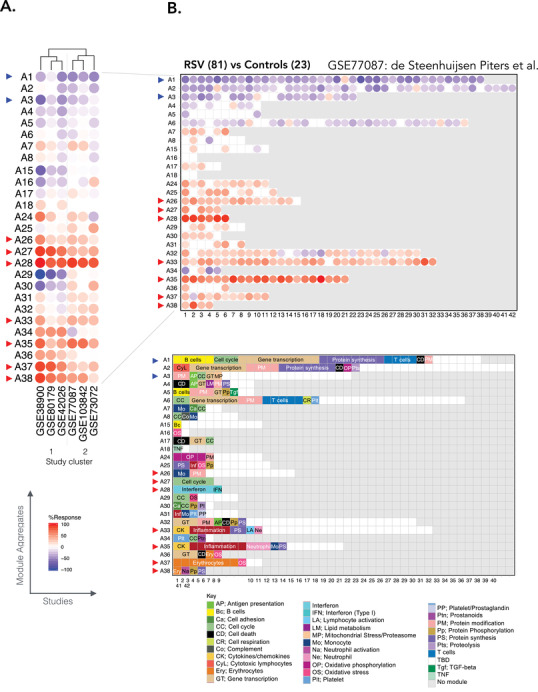
Modular repertoire changes in patients with respiratory syncytial virus (RSV) infection versus uninfected controls. A, Fingerprint heat map comparing the module aggregate level patterns of transcript abundance across six RSV datasets. The summarized module aggregate level values on this heat map are arranged in rows and the datasets in columns. The datasets are grouped via hierarchical clustering, according to similarities in patterns of transcript abundance across module aggregates. B, Fingerprint grid for GSE77087. Modules are assigned a fixed position on the grid, with each row corresponding to a “module aggregate” constituted of modules following similar patterns of change in transcript abundance. The number of constitutive modules for each aggregate ranges from two (A16) to 42 (A2). Aggregates comprising a single module are not represented on this map (A9‐A14; A19‐A23). The percentage of constitutive transcripts for a given module showing an increase in abundance in RSV patients over controls is indicated by a red spot. The percentage of constitutive transcripts showing a decrease in abundance for a given module is indicated by a blue spot. The color key at the bottom indicates the functions that have been associated with some of the modules on the grid

Here, we mapped the changes in transcript abundance for each dataset against this modular framework. This process consists of determining the percentage of transcripts constituting a given module that is significantly changed. This procedure is repeated in turn for each module and across each one of the six datasets comprised in our collection. This approach made it possible to assess, as a first step, the degree of consistency in the RSV response signatures across the datasets. To facilitate interpretation, we represented the changes at the least granular level by showing on a heat map the abundance profiles for each of the six RSV datasets (columns) across 27 module aggregates (rows) (Figure 1A). From this highly reduced set of variables, we could pinpoint the most conserved molecular signatures across the six datasets. These included seven aggregates showing consistent increases in transcript abundance (observed in at least 5/6 datasets: A26, A27, A28, A33, A35, A37, A38), and two aggregates showing consistent decreases (A1, A3). We also observed changes for another set of modules but in only three of six RSV datasets (A15, A16, A29, A30, A34, A36). Technical or biological parameters (Table 1) did not yield an obvious explanation for the differences between these two groups of studies. The amplitude of the changes in other aggregates was minimal. Notably, the dataset from Liu et al (GSE73072^21^) that comprised adults infected experimentally with RSV did not behave as an outlier in this analysis. At the module aggregate level patterns of transcript abundance was most similar to that of two pediatric datasets (GSE103842, GSE77087), which together clustered away from the remaining three pediatric datasets (Figure 1A).

Taken together, this step permitted the mapping of transcriptional changes measured across different RSV datasets using the same transcriptional module framework. This was useful in relating changes observed between the studies and pinpointing signatures that seem to be most robustly associated with RSV infection.

### 2.3 Changes in transcript abundance can be mapped to a fixed transcriptional module repertoire to facilitate functional interpretation

To functionally interpret the conserved signatures observed across RSV datasets, a more granular level of information is needed. We thus examined transcriptomic changes at the level of the modules forming the 27 aggregates mentioned above. We represented the changes in transcript abundance as grid plots for each of the RSV datasets (Figure 1B, Figure S1). A first vertical reading of the grid across the rows provides a sense of the changes at the aggregate level already summarized in the heat map that was presented earlier (Figure 1A). A second horizontal reading across the columns provides a sense of the changes occurring at the module level within each of the aggregates.

Because the positions on the grid are fixed, it is possible to overlay other information, such as functional annotations (color‐coded grid in Figure 1B). We found that some of the conserved signatures that were increased during RSV infection comprised modules preferentially associated with interferon responses (A28), inflammation (A33, A35), erythrocytes (A37, A38), and cell cycle (A27), while those that were decreased were associated with lymphocytic responses (A1, A3). Some of these responses are further interpreted below, and all details are accessible via interactive web presentations for modules constituting each aggregate (web links are listed in Table 2). The presentations include reports from functional profiling analyses carried out using different tools. Heat maps representing the patterns of abundance for transcripts constituting each module across reference datasets are also available. Furthermore, a dedicated web application was developed in support of the work presented here and permits users to access the fingerprint grid plots and to generate other types of plots that are presented throughout this manuscript. This resource can be accessed via this link: https://drinchai.shinyapps.io/RSV_Meta_Module_analysis/. A video demonstration can be accessed here: https://youtu.be/htNSMreM8es.

**TABLE 2:** Links to module aggregates annotation pages

**Aggregate**	**Function**	**Link**
A1	Lymphocytic	https://prezi.com/view/sxap39tKxkmCNTTNIlVO/
A2	TBD	https://prezi.com/view/96GWajx5mZjuRS4B6gjA/
A3	TBD	https://prezi.com/view/OWFVI51FND0WWwNgsgJZ/
A4	TBD	https://prezi.com/view/2Zbq8ZDYbO4hbUd4r2KF/
A5	Lymphocytic	https://prezi.com/view/62tgA5E6roOlk5DRNvS1/
A6	Lymphocytic	https://prezi.com/view/Uks2Nd4lvizNNFVPtBEy/
A7	TBD	https://prezi.com/view/kKfergNj0SkLXyFtm0Dg/
A8	TBD	https://prezi.com/view/Y4uk1RPJyNcSndJYnFX6/
A15	TBD	https://prezi.com/view/jgYehQ9QhyADAttEsdoI/
A16	TBD	https://prezi.com/view/SKzHeA0XYdLYvy2sY8gP/
A17	TBD	https://prezi.com/view/FS7sE1Vqew5g8EKOM1AM/
A18	TBD	https://prezi.com/view/aZMLflMNVrV7JnVaIILm/
A24	Oxidative phosphorylation	https://prezi.com/view/eiXvf2LNBLFRgrtaeCuM/
A25	TBD	https://prezi.com/view/pwyojaU62Z7GT102ZYwM/
A26	TBD	https://prezi.com/view/9CErpW3NwpN2HgRS3Hzf/
A27	Cell cycle	https://prezi.com/view/GgIiA0K9kSFHbpVj2I85/
A28	Interferon	https://prezi.com/view/E34MhxE5uKoZLWZ3KXjG/
A29	TBD	https://prezi.com/view/W4TShTd32dEJx0XPOF1U/
A30	TBD	https://prezi.com/view/kl7VHoJTWug0sn7TgXut/
A31	TBD	https://prezi.com/view/GqtUO22JJlSf16zMJKbB/
A32	TBD	https://prezi.com/view/qlbG9VFzegOndQKD8aiy/
A33	Inflammation	https://prezi.com/view/VBqKqHuLWCra3OJOIZRR/
A34	TBD	https://prezi.com/view/HcSgIEGP3TJjTSpaPCxv/
A35	Inflammation	https://prezi.com/view/7Q20FyW6Hrs5NjMaTUyW/
A36	Erythroid	https://prezi.com/view/M7dnztl2h61gXrKFQeB2/
A37	Erythroid	https://prezi.com/view/YyQs4WiXSNf0YXE79lfS/
A38	Erythroid	https://prezi.com/view/0KUlPICKUZGeUjb206R5/

In summary, we mapped the conserved RSV signatures identified to a well‐annotated modular framework. This mapping made it relatively straightforward to assign each signature to a predetermined functional category. The added granularity and available online resources make it possible to further dissect these signatures in subsequent analyses, as we exemplify below.

### 2.4 Blood transcriptional signature presents a high level of interindividual variability among cohorts of RSV patients

Blood transcriptome profiling provides a means to measure interindividual differences with a high degree of resolution. Understanding the biological and clinical significance of this variability is important but requires the study of large patient cohorts. The collective reanalysis of the six datasets assembled here provides a unique opportunity to investigate interindividual differences among patients with RSV infection at the molecular level.

**FIGURE 2:**
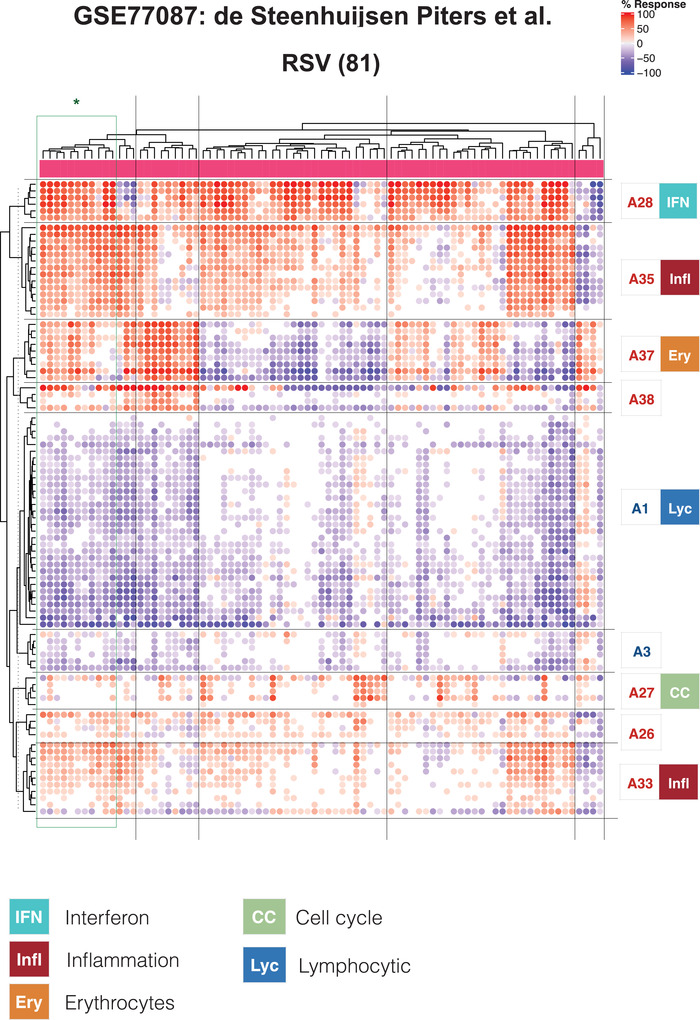
Heat map representation of changes in abundance of transcriptional modules across respiratory syncytial virus (RSV)‐infected individuals. This heat map was generated for the GSE77087 dataset that is also represented as a fingerprint grid plot in Figure 1B. The modules comprised in aggregates identified as being conserved (indicated by the colored triangles in Figure 1A) are arranged as rows, and the RSV subjects comprised in this dataset are arranged as columns. The colored spots represent the percentage of transcripts within each module deemed to be differentially expressed (up = red, down = blue). The modules are arranged based on similarities in abundance patterns via hierarchical clustering within each aggregate. A general function is attributed to some of the aggregates, as indicated by the colored symbols and key

The approach that we used next to map changes in transcript abundance for individual patients against the repertoire of transcriptional modules is very similar to that described above for groups of patients. We expressed the changes for each individual RSV patient as a percentage of the constitutive genes for which the abundance was increased or decreased compared to the respective control group (see Section 4 for details). As an illustration, we generated a heat map (Figure 2) of the results obtained for subjects comprising the de Steenhuijsen Piters dataset (Figure 1B). The patterns in the changes in abundance are only shown for the modules constituting the nine aggregates deemed to be conserved across the collection of RSV datasets (highlighted in Figure 1). We generated similar plots for each of the remaining datasets (Supporting File, and https://drinchai.shinyapps.io/RSV_Meta_Module_analysis/).

From the heat maps, we observed that interindividual variability exists even for signatures that at the group level were well conserved across the six datasets (Figure 1A). In reality, only a minority of patients in this illustrative dataset (11/81) matched the prototypical pattern defined above at the group level based on conserved changes observed for nine module aggregates (i.e., A1− A3− A27+ A28+ A33+ A35+ A37+ A38+; Figure 2). This finding highlights the importance when conducting such analyses of delineating the extent and nature of interindividual variations that may exist among patients. Here, the degree of interindividual variability differed from one module aggregate to another. For example, in the modules forming aggregates A1 (lymphocytic) or A33 (inflammation), changes in abundance only varied in amplitude without, for the most part, showing an inversion of trends. In other modules, inverted trends were much more common, as exemplified by A37 (erythrocytes).

Taken together, examining the changes in transcript abundance at the level of individuals revealed a significant degree of heterogeneity among cohorts of RSV patients. This paradigm was also true for signatures deemed to be conserved when carrying out comparisons at the group level.

### 2.5 Distinct blood transcriptome phenotypes are identified among a consolidated cohort of RSV patients

The fact that the consensus disease signature defined earlier was not reflected at the level of individual patient, highlighted the need to characterize distinct RSV blood transcriptome phenotypes. For this, we used the combined set of patients from the six public transcriptome datasets. First, we generated PCA plots to evaluate the sources of variance among this composite set of samples (Figure S2A). The results indicated the absence of study bias when abundance measures were normalized to the respective control group and reduced to module level summarized values. This finding was largely confirmed when representing interindividual differences on a tSNE plot (Figure S2B).^23^ One dataset did show partial separation from the others, but this only concerned a minority of subjects and could also be attributable to biological sources. The same shift was observed on the PCA plot but only along PC3, which accounts for only 9% of the overall variance.

Next, we selected parameters (or “traits”) that would be used for defining the RSV blood transcriptome phenotypes. As a first step, we sought to identify the signatures with the highest degree of interpatient variability, assuming that these would be best able to discriminate patients according to phenotypes. We identified these signatures at the least granular, module aggregate level, thus starting from a set of only 38 variables (Figure S3). We selected the following four aggregates: (a) A27, comprising five modules functionally associated with the “cell cycle”; (b) A28, comprising six modules functionally associated with the “interferon response”; (c) A35, comprising 21 modules predominantly associated with “inflammation”; and (d) A37, comprising 11 modules predominantly associated with “erythrocytes.” We did not include two other module aggregates that exhibited a similar degree of interpatient variability, but which at a high level exhibited a notable degree of functional convergence with the four selected aggregates. The two aggregates that we did not include are: A33, which like A35 is also associated with “inflammation”; and A38, which like A37 is associated with “erythrocytes.” Limiting the number of parameters to only four helped keep the number of possible phenotypes to a reasonable level (81 in total) given the sample size (N = 319 RSV patients). Notably, larger study cohorts should accommodate the definition of more complex phenotypes (i.e., selection of more than four parameters, or “traits”).

**FIGURE 3:**
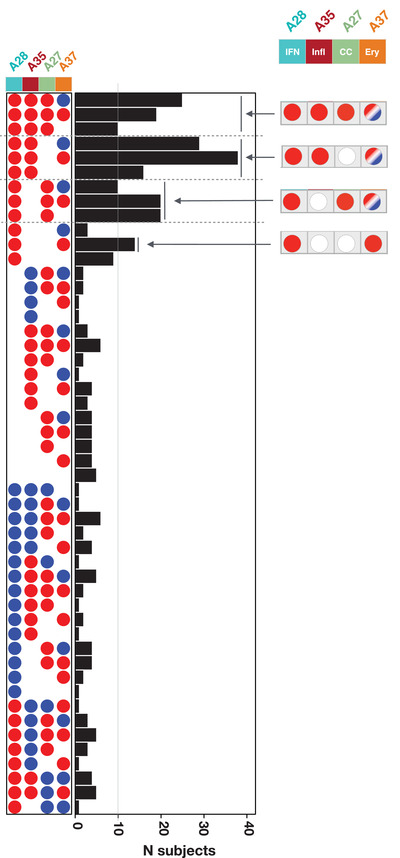
Stratification of respiratory syncytial virus (RSV) patients according to blood transcriptome phenotypes. Phenotypes were defined according to four different “traits.” Each of the 319 RSV patients comprising the consolidated cohort used in this meta‐analysis was assigned to a phenotype according to their status for each of the four traits: positive (red), negative (blue), or unchanged (white). This determination was made in reference to each respective control baseline. The bar graph shows the number of patients assigned to each of the phenotypes. The gray line indicates the threshold used to select the phenotypes considered to be the most abundant (>10 subjects)

Next, we assigned the status for each aggregate signature in a given individual using the corresponding percentage of increased or decreased transcripts: if the value was >15% the aggregate was considered to be increased (noted as +), if <15% it was considered to be decreased (noted as −), else it was considered not changed (noted as 0). An example of such a notation for a given phenotype would be Infl0/IFN+/CC+/Ery−. We then generated the distribution of subjects constituting the combined RSV cohorts across all 81 possible Infl/IFN/CC/Ery phenotypes (Figure 3). We found that a small subset of phenotypes comprised a higher number of patients than others (>10 per phenotype). These phenotypes were all positive for the “interferon” trait (IFN+), were positive or showed no change for the “inflammation” and “cell cycle” traits (Infl+/0 or CC+/0), and exhibited any erythrocyte trait status (Ery+/0/−). Phenotypes where the interferon status was unchanged or decreased were comparatively less prevalent (five patients per group at most), and so were phenotypes where interferon was increased but inflammation or cell cycle phenotypes were decreased.

Overall, we showed that a principled approach using a small subset of highly reduced variables can identify a discrete number of interpretable RSV blood transcriptome phenotypes.

### 2.6 A subset of RSV blood transcriptome phenotypes is associated with severe disease

An obvious next question is whether the stratification of RSV patients according to blood transcriptome phenotypes, such as those described above, has any clinical relevance. The extent of phenotypic information made available alongside the blood transcriptome datasets varied greatly between studies. Notably, pertinent information reflecting disease severity (eg, respiratory rate, transcutaneous O_2_ saturation) was lacking for many patients. As a result, we had to use a relatively crude metric of disease severity that relied on the type of care the patient required; that is, whether they were outpatients, inpatients cared for in the ward or were admitted to the pediatric intensive care unit (PICU).

**FIGURE 4:**
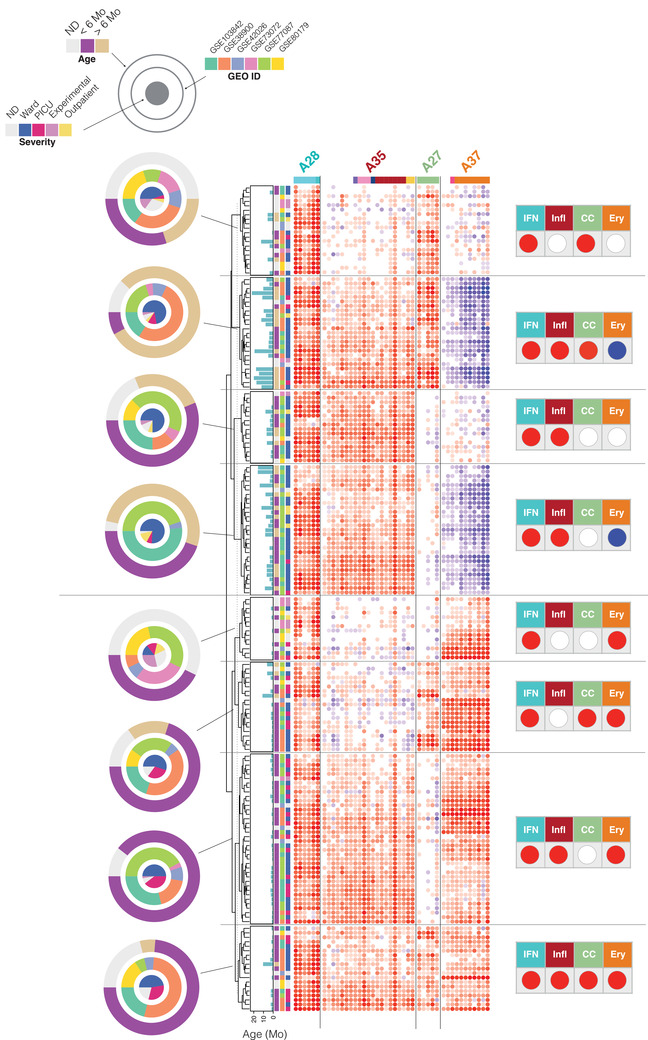
The association of dominant respiratory syncytial virus (RSV) blood transcriptional phenotypes with clinical and demographic attributes. Heat maps were generated for the seven most prevalent phenotypes identified on the distribution plot. The subjects (rows) were first arranged according to their phenotype, and then arranged within each phenotype according to similarities in abundance patterns. Modules constituting the four aggregates selected for the definition of molecular phenotypes are shown as columns. The respective traits for module aggregates A28, A35, A27, and A37 are IFN (interferon), Infl (inflammation), CC (cell cycle), and Ery (erythrocytes). The status for each phenotype is indicated by a red, blue, or white spot (increase, decrease, or no change, respectively). The concentric circle plots (right) indicate the distribution of patients constituting each phenotype according to age, study membership, and RSV severity status.

We first visualized the patterns of transcript abundance at the module level for individuals belonging to the eight most prevalent phenotypes (Figure 4). This heat map verified that the phenotypic categories presented a high degree of homogeneity. Upon overlaying the phenotypic information on this plot, we gained first indication of a possible association between age and disease severity. Specifically, it was possible to discern a trend toward a younger age among Ery+ subjects in comparison to Ery− or Ery0 subjects. Importantly, the different studies also seemed to be well represented in each of the phenotypes, indicative of their underpinnings by biological rather than technical factors.

**FIGURE 5:**
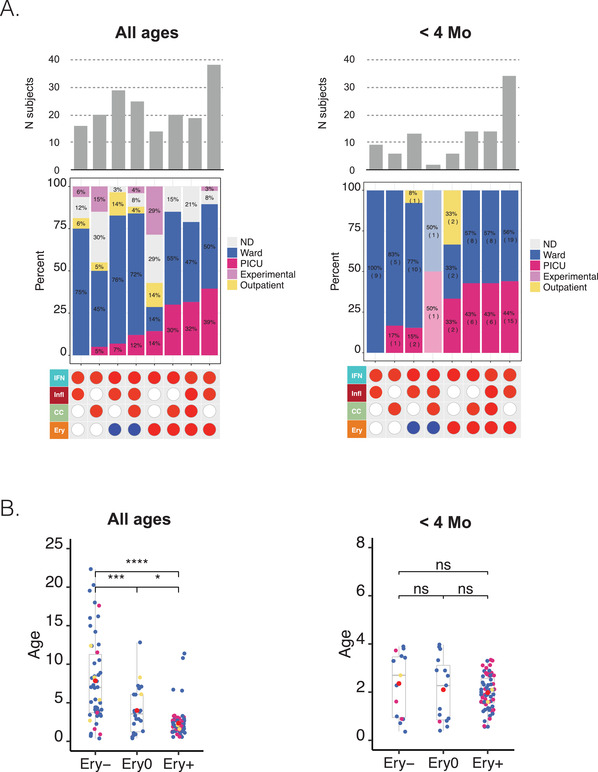
The association of dominant respiratory syncytial virus (RSV) blood transcriptional phenotypes with disease severity as a function of age. A, Bottom panel: The red or blue spots define the status for four traits corresponding to the eight most prevalent RSV phenotypes identified in Figure 4 (>10 subjects). Middle panel: The relative frequency of subjects cared for in the hospital ward, PICU, outpatients, and experimentally exposed subjects. This information was not available for all studies. Top panel: Bar graph showing the number of subjects comprising each of the eight different phenotypes. The combination of tables and graphs on the left is for all subjects. Information for a subset of subjects <4 months is shown on the right. B, The box plots represent the age in months of individuals comprising the consolidated RSV cohort used throughout this study. Patients were categorized according to their Ery trait status. The plot on the right shows the same information but of a subset of patients <4 months. Dots are color‐coded according to severity status (red = PICU, blue = ward, yellow = outpatient). *P < .05, **P < .01, ***P < .001

We then looked at the relative proportion of severe patients for each high‐prevalence phenotype and the contributions by the different studies (Figure 5A). Four of the eight phenotypes were Ery+, of which three comprised a proportion of PICU patients that was on average 5.6 times higher than the four Ery− and Ery0 phenotypes. The Ery+ patients included the quadruple positive IFN+/Infl+/CC+/Ery+ phenotype with 32% of PICU patients, while its IFN+/Infl+/CC+/Ery− counterpart had 12% of patients. We found no severe patients in the IFN+/Infl+/CC0/Ery0 group. Furthermore, subjects with Ery+ phenotypes were significantly younger than subjects with Ery− phenotypes (*P* < .001, Figure 5B).

We next wanted to determine whether the presence of the Ery trait was associated with heightened severity, regardless of age. For this, we examined the distribution of severe cases across the same phenotypes but focused on infants <4 months old (Figure 5A). This cutoff was chosen because no associations between age and Ery levels were observed for this age group (Figure S4), whereas an association was evident among subjects <6 months old (a cutoff that is more customary in immunological studies and was employed in some of the analyses presented in the original papers). We found that among infants <4 months old, the severe cases were again distributed preferentially among Ery+ phenotypes ([Ery+ = 29 PICU] cases, Ery−/0 = 4 PICU cases; of note, the IFN+/Infl+/CC+/Ery− phenotype comprised only two patients, one of which was a PICU case).

**FIGURE 6:**
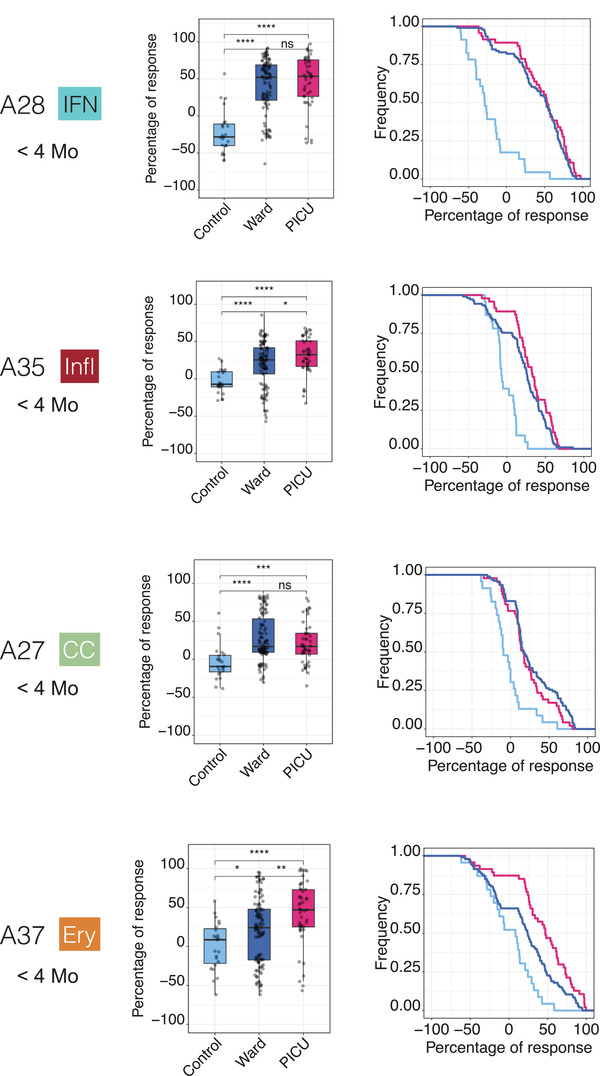
Association of blood transcriptomic traits with respiratory syncytial virus (RSV) disease severity in infants <4 months of age. Box plots (left) show levels of transcript abundance measured for individual subjects for a given aggregate. This value represents the percentage of transcripts constituting the aggregate that is increased or decreased for an individual compared to the median of the uninfected control group (+100% = all transcripts are increased; −100% = all transcripts are decreased). Individuals are grouped according to health status: uninfected controls, inpatients cared for in the hospital ward, or inpatients cared for in the PICU. *P < .05, **P < .01, ***P < .001. The “Signature survival” curves (right) represent the relative frequency of subjects (*y*‐axis) for whom the percentage response falls at or above a given threshold (*x*‐axis). The percentage response was calculated in the same manner as described for the box plot. Thus, all subjects would have a percentage of response falling between −100% and +100%, as indicated by the curves showing the frequency values of 1 at *x* = −100%. As the range narrows, the frequency decreases; in most cases a very small proportion of patients have a percentage of response values falling between +90% and +100% (at x = +90%). The separation of the curves is an indication of the differences in the distribution of percentage responses between groups (pale blue = control, dark blue = ward, red = PICU).

Finally, we investigated the associations between each of the four traits used for RSV patient phenotyping and stratification, and disease severity (Figure 6). Here, we found that the abundance of transcripts forming the A37/erythrocyte cell aggregates were significantly increased in patients cared for in the PICU compared to the ward (Ery trait; *P* < .01). We made a similar finding for the A35/“inflammation” aggregate, although to a lesser degree (Infl trait; *P* < .05). We found no significant differences for the A27/cell cycle or A28/interferon aggregates. Associations can be explored for various aggregates as a function of age via our web application (https://drinchai.shinyapps.io/RSV_Meta_Module_analysis/).

Taken together, these findings suggest that our RSV stratification system might be clinically relevant. This conclusion is illustrated by the fact that a high proportion of severe subjects was observed among most phenotypes positive for the Ery trait. This finding might be particularly relevant in infants <4 months old who would otherwise carry a similar risk of developing severe RSV disease when taking age into consideration.

### 2.7 The RSV “erythrocyte” signature is shared with melanoma patients and liver transplant recipients

Beyond the question of clinical relevance of these RSV blood transcriptome phenotypes, we next sought to understand their biological significance. For this we relied on several resources. First, we used a web application providing access to a reference collection of module fingerprints for the 16 pathological or physiological states^19^ (accessible via: https://drinchai.shinyapps.io/dc_gen3_module_analysis/#; demonstration video: https://youtu.be/y__7xKJo5e4).

**FIGURE 7:**
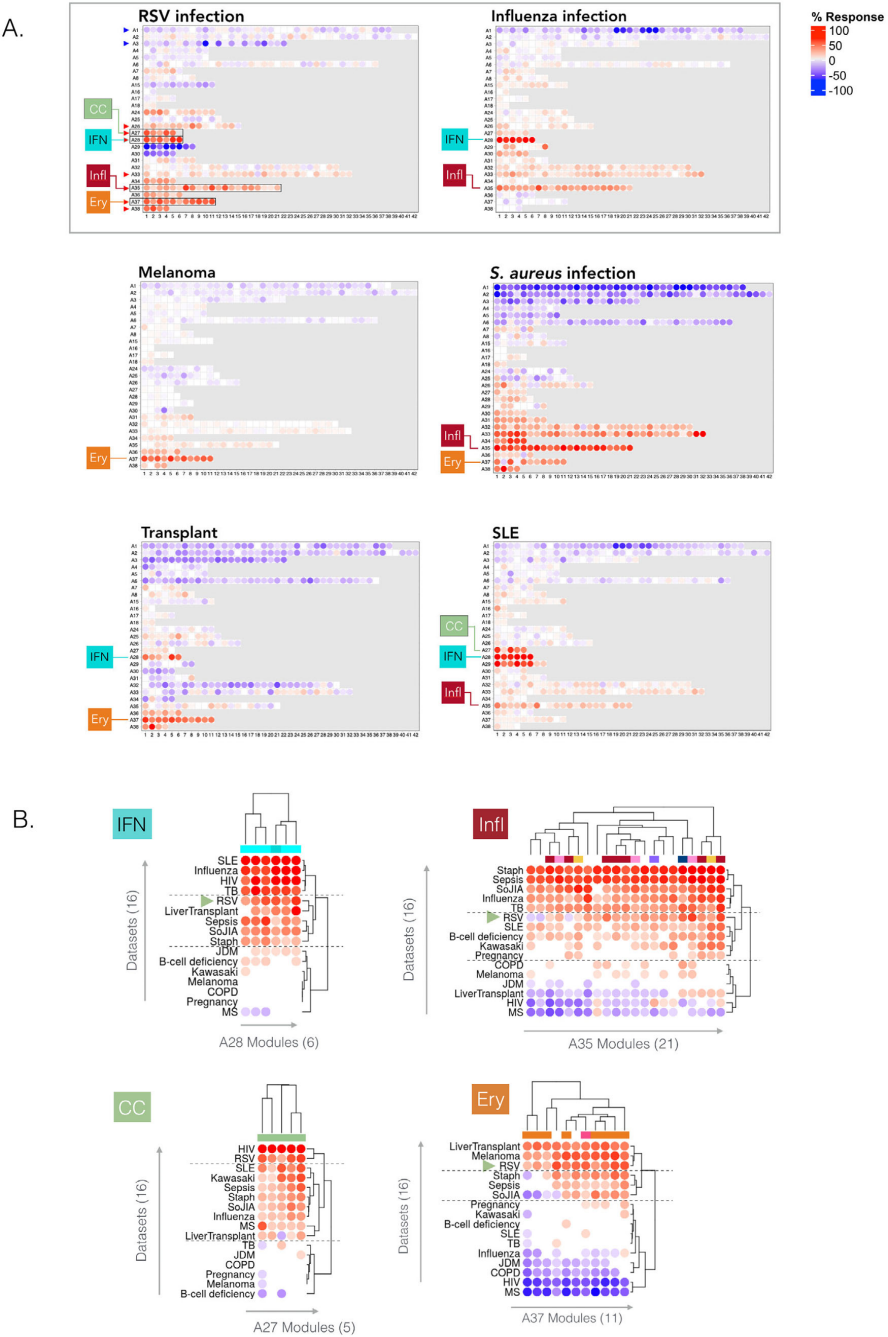
Contextual interpretation of respiratory syncytial virus (RSV) blood transcriptome fingerprints A, Fingerprint grid plots displaying changes in the levels of transcript abundance in patients with RSV infection, influenza infection, systemic lupus erythematosus, stage IV melanoma, or in liver transplant recipients. The visualization scheme is similar to the one described for the fingerprint grid plot in Figure 1B. The four traits used to molecularly stratify RSV patients are highlighted. Because position of the modules on the grid is fixed, the color key from Figure 1B can also be used for functional interpretation of the modules from the other rows. The datasets on which the fingerprint maps are based are publicly available under Gene Expression Omnibus (GEO) accession ID GSE100150. B, Heat maps displaying the changes in transcript abundance for modules belonging to four aggregates (columns) across 16 reference datasets. As for the grid plots, an increase and decrease in the abundance of transcripts constituting these modules are shown by a red or blue spot, respectively. The rows (datasets from each disease cohort) and columns (modules) were arranged by hierarchical clustering based on similarities in patterns of transcript abundance. All the plots can be generated and exported via a web application: https://drinchai.shinyapps.io/dc_gen3_module_analysis/#; video demonstration: https://youtu.be/y__7xKJo5e4

We generated fingerprint grid plots to compare the changes in transcript abundance in acute influenza and RSV infections (Figure 7A). Acute influenza infection highly resembles RSV infection in terms of clinical presentation, especially in infants. As could be expected, the fingerprints of both of these respiratory infections featured a potent interferon signature (modules in aggregate A28; ie, the IFN trait defined earlier). The modules associated with inflammation (comprised in aggregate A35; Infl trait) were also generally increased in both diseases. However, one of the most marked differences between the influenza and RSV fingerprints concerned the erythrocyte signature (aggregate A37; Ery trait), which was consistently increased in RSV but was unchanged in influenza.

Another fingerprint dominated by an interferon signature was that of systemic lupus erythematosus (SLE), but as was the case for influenza, it did not comprise an “A37/erythrocyte” signature. Among the fingerprints of other reference datasets, those of *Staphylococcus aureus* infection, liver transplantation, and metastatic melanoma also showed an elevation in abundance for transcripts constituting modules belonging to the A37 aggregate (Figure 7A). The *S. aureus* infection fingerprint showed the highest degree of alteration overall, with widespread changes in transcript levels occurring in most aggregates. This finding contrasts with the fingerprint for metastatic melanoma, which for the most part was quiescent, except for a marked increase in the abundance of genes constituting the A37 modules. The signature observed in liver transplant recipients receiving maintenance immunosuppresive therapy was more perturbed than that of melanoma patients, but it was likewise characterized by an increase in the abundance of transcripts constituting the “A37/erythrocyte” modules.

Next, we used the same web application to examine module abundance profiles specifically for the IFN, Infl, CC, and Ery traits across all 16 reference datasets (Figure 7B). For the IFN trait (A28), RSV clustered among the diseases showing an intermediate level of response, along with liver transplant recipients, patients with systemic onset juvenile idiopathic arthritis (SoJIA), *S. aureus* infection (pediatric), or sepsis caused by various pathogens (adults). Influenza was clustered among diseases showing the highest IFN responses, including other infections such as tuberculosis or HIV, as well as SLE (Figure S5). For the Infl trait (A35), RSV clustered again with diseases showing an intermediate response level, and predictably lower than those measured not only in SoJIA, sepsis, and *S. aureus*, but also influenza infection. For the CC trait (A27), the RSV and HIV cohorts formed a cluster with the highest increase in abundance. Finally, for the Ery trait (A37) the RSV cohort was one of only three diseases in the high‐abundance cluster, along with the melanoma and liver transplant cohorts. We observed increases to a lesser extent in diseases characterized by overt systemic inflammation, such as sepsis or SoJIA, as well as in pregnant women. This trait tended to be decreased in other viral illnesses, such as influenza and HIV infection.

Overall, this contextual interpretation of the dominant traits comprising the RSV signature identified some peculiarities. Notably, the interferon response that, while robust, seemed to be somewhat muted when compared to other viral infections. More striking was the atypical overall elevation in the abundance levels associated with the erythrocyte signature. The extent of the observed change was only found in melanoma patients and liver transplant recipients: in these two cohorts, the erythrocyte signature dominated the overall changes observed in the blood transcriptome. Notably, both patient populations relate to states characterized by marked immunosuppression, driven by the disease in the first case and pharmacological treatment aiming at maintaining graft tolerance in the other.

### 2.8 Expression of transcripts constituting the A37/“erythrocyte” modular signature is restricted to a population of fetal GlyA+ erythroid cells

**FIGURE 8:**
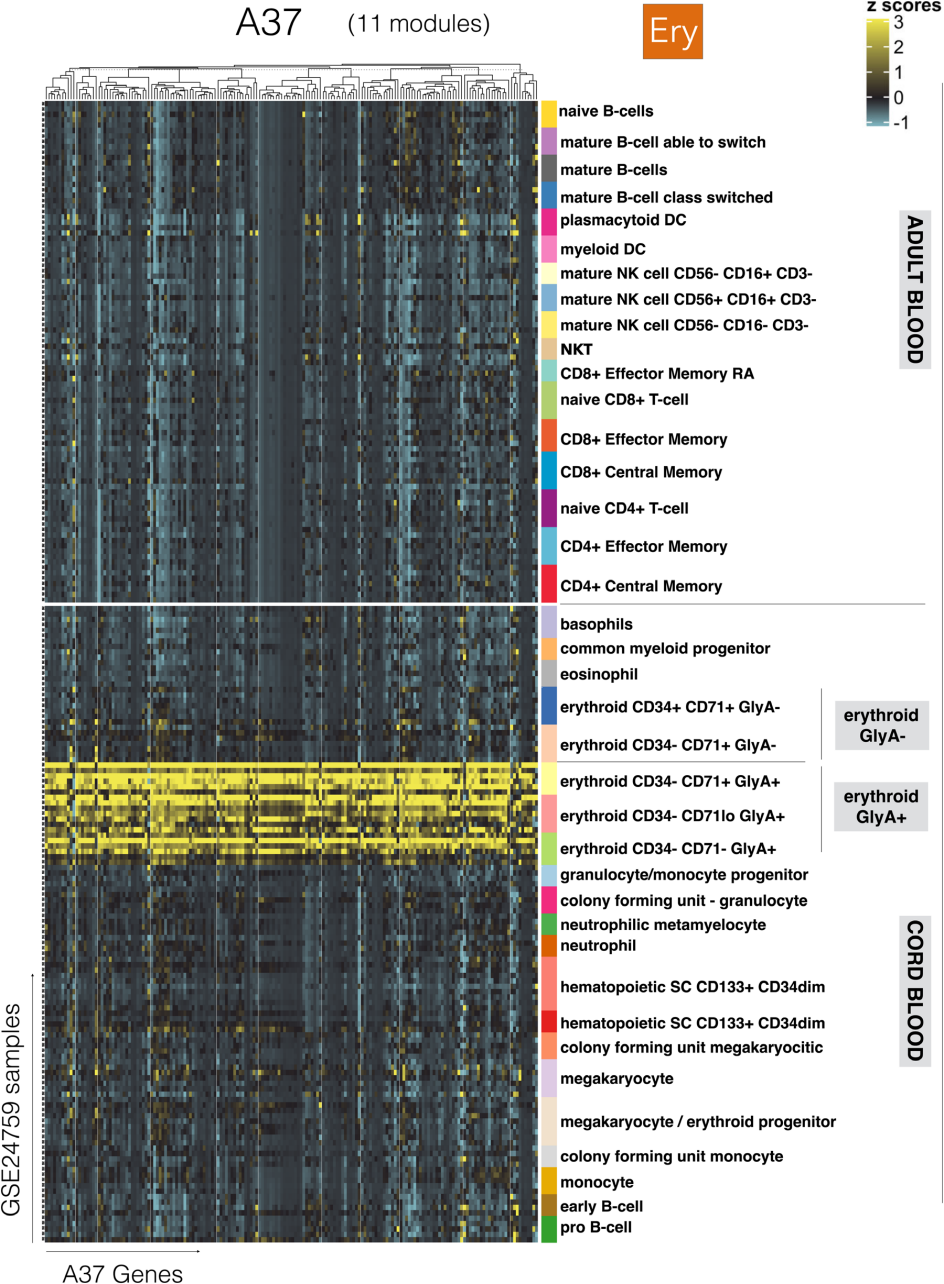
Expression levels of A37 genes across cell populations isolated from human peripheral blood and cord blood. The abundance levels of transcripts comprised in the 11 modules constituting A37 (columns) across blood‐cell populations (rows). The dataset is publicly available under GEO accession ID GSE24759 (24). The populations are separated based on whether they were isolated from adult venous blood (top) or from neonate cord blood (bottom). Distinct erythroid cell populations isolated on the basis of cell surface expression of CD34, CD71 and GlyA antigens are also shown

In our final analyses, we focused our interpretations on the erythrocyte signature (A37). Although we had observed an association with RSV disease severity, we did not ascertain causality. Based on functional profiling results that were run using multiple approaches, we attributed the erythrocyte annotation to 8/11 modules in aggregate A37 (interactive reports available via: https://prezi.com/view/YyQs4WiXSNf0YXE79lfS/). Examining the abundance patterns for the transcripts comprising the A37 modules in reference datasets comprising isolated leukocyte cell populations provided further insight (Figure 8; with additional heat maps accessible via the weblink provided above). In one such reference dataset contributed by Novershtern et al,^24^ the expression of A37 transcripts was narrowly restricted to populations of glycophorin A‐positive (GlyA+) fetal erythroid cells. This pattern was irrespective of CD71 marker expression. However, genes comprising A37 modules were not expressed in CD71+ but GlyA− cells. We observed similar expression patterns for modules constituting aggregates A36 and A38, both comprised one module functionally associated with the erythrocyte annotation (Figures S6 and S7).

Erythroid precursors of fetal origin can circulate in the blood of neonates for up to 3‐4 weeks following birth. Immunosuppressive properties have been attributed to these populations^25^; for example, this cell population confers susceptibility to Listeria infection in neonates.^26^ However, a possible role for these circulating erythroid cells (CECs) in the context of RSV infection has not been investigated to date. Others have also described the presence of an erythroid cell population with potent immunosuppressive properties associated with anemia in adults with late‐stage cancer.^27^ This finding is consistent with our observation of a prominent A37/erythrocyte signature in the melanoma patients included in our study.

Taken together, these observations support the notion that an increase in A37 transcript abundance is associated with the presence of CECs. These cells might possess immunosuppressive properties and conceivably contribute to the worsening of RSV infection. While these findings might be particularly relevant to young infants, we also observed increases in adult subjects exposed experimentally to the virus (GSE73072: Figure 1A, Figure S1).

## 3 DISCUSSION

This work has built on earlier studies investigating host responses to RSV via blood transcriptome profiling. The approach we adopted did not focus on identifying sets of classifiers or predictors. Rather, we primarily documented the interindividual differences among this large, consolidated set of patients. Relying on highly reduced dimensions made it possible to define dominant blood transcriptome phenotypes among this aggregated RSV patient cohort. The four “traits” or signatures that were retained included: interferon (A28 aggregate/IFN trait), inflammation (A35/Infl), cell cycle (A27/CC), and erythrocytes (A37/Ery). Out of 319 RSV subjects, 199 were distributed in just eight phenotypes out of a possible 81. These dominant phenotypes were all positive for the interferon trait and positive or unchanged for the inflammation and cell cycle traits. The erythrocyte trait status ranged from being increased, unchanged, or decreased.

From the standpoint of clinical significance, the phenotypes positive for both the interferon and erythrocyte traits were generally associated with a higher proportion of severe patients. However, the levels of increase in abundance of interferon‐inducible transcripts did not correlate with disease severity. Rather, erythrocyte transcripts showed the strongest association with infection severity. This finding confirmed (on a larger scale) the association previously described by Mejias et al, which was based on the analysis of one of the datasets comprised in this collection.^7^ We also observed an association, although to a lesser degree, between the level of increase of transcripts forming an inflammation signature (aggregate A35) and infection severity. Again, this is in line with previous findings.^9^, ^10^ The approach that we employed here permitted the integration of such signatures into more complex phenotypes as well as individual benchmarking/prioritization in a much larger aggregated cohort of patients. Follow‐up prospective studies are now warranted to validate and maybe further refine such classification scheme. For instance, the number of traits needed for this classification to be clinically relevant would have to be determined. Thus far, our analysis suggests that the erythrocyte trait might constitute a valuable risk indicator if testing focuses on a narrowly defined age group (eg, <4 months of age).

Our meta‐analysis of available RSV blood transcriptome datasets also yielded several insights relevant to the immune biology of this disease. The interferon signature (A28/“IFN” trait) is a hallmark of RSV infection and is observed in a wide range of other viral and bacterial infections as well as autoimmune diseases, as illustrated in the set of 16 blood transcriptome datasets used in our interpretation. Our previous work has suggested that subsets of modules constituting this aggregate are preferentially induced by type I interferons (M8.3, M10.1).^19^ Consistently, we also observed changes for these modules in patients with RSV infection (Figure S5). Others have also described robust interferon responses as measured via blood transcriptome profiling in RSV patients.^11^ The role of type I interferons per se is not widely reported, but a recent study describes a dependency on interferon alpha and beta for developing antibody‐mediated responses.^28^ Several reports, however, have identified reduced interferon gamma responses in the context of RSV infection, especially when compared to the response observed during influenza virus infection.^29^, ^30^, ^31^, ^32^ This finding is also consistent with our observation of a somewhat muted interferon response in RSV patients compared to what we measured in response to not only influenza, but also to HIV or TB infection, as well as in patients with SLE.

We propose that the inflammation signature (A35/“Infl” trait) is associated with “neutrophil‐driven” inflammation, given the preferential expression of its constitutive transcript in neutrophils and induction in patients with sepsis. This information is derived, in part, from a dataset contributed by Linsley et al that comprised RNAseq profiles of isolated leukocyte fractions.^33^ The patterns of transcript abundance are available for the modules constituting aggregate A35 via an interactive web presentation (https://prezi.com/view/7Q20FyW6Hrs5NjMaTUyW/). This aggregate was also the focus of a recent reanalysis that we conducted, in which an increase in abundance of A35 transcripts was a dominant feature of the psoriasis blood transcriptome fingerprint.^34^ In this work, we hypothesized in turn that this inflammatory response might be driven more specifically by interleukin‐17 (IL17). And indeed, several researchers have found a role for IL17 in the context of RSV infection,^35^, ^36^ and in one instance specifically indicating the involvement of neutrophils in IL17‐mediated antiviral responses.^37^

We posit that the cell cycle signature (A27/“CC” trait) is associated with the expansion of plasmablasts, which are responsible for antibody production. Indeed, modules in this aggregate comprised an overabundance of genes involved in the cell cycle, such as cyclins. One of the modules also comprises several genes expressed by plasmablasts (M12.15: CD38, IGJ, TNFRSF17).^38^, ^39^, ^40^ Notably, we have reported earlier that abundance levels of those transcripts is also markedly increased between 7 and 14 days after the administration of trivalent influenza or pneumococcal vaccines.^41^ In the context of this earlier study, changes in the abundance of these markers were shown by flow cytometry to correlate with the presence of antibody‐producing cells. These levels also correlated with antibody titers measured 4 weeks postvaccination. Indeed, antibody‐producing cells also expand during the course of RSV infection. Habibi et al reported a peak in antibody‐producing cells, 10 days after experimental exposure to RSV as well as a correlation with the levels of neutralizing antibodies developed by the study subjects.^42^

Consistent with our earlier findings,^7^ the erythrocyte signature (A37/“Ery” trait) was most strongly associated with RSV infection severity. We putatively link this signature with the presence of CEC precursors, based on the restriction of A37 transcripts in a reference transcriptome dataset to fetal erythroid cells (Figure 8). Erythroid precursors would normally be found in the bone marrow, but cells originating from the fetal liver do persist in infants in the circulation for a few weeks after birth.^43^ In adults, extramedullary erythropoiesis is observed in the spleen and liver, and occurs under various circumstances, including anemia, pregnancy, severe infection, or chronic stress.^44^, ^45^ Indeed, in the context of the present study, it is worth noting again that an increase in abundance of A37 transcripts was also observed in adult subjects experimentally infected with RSV (GSE73072 dataset, from Liu et al^21^). We hypothesize that the CECs associated with this signature might have immunosuppressive functions during an RSV infection. Thus, CEC‐mediated immunosuppression would in turn drive a worsening of disease and severity in patients with RSV infection. This assertion is supported by various lines of evidence. First, Elahi described a wide range of mechanisms conferring immunosuppressive properties to this cell population, including via cell surface receptors (such as PD1/PDL‐1 and VISTA) or soluble factors (such as arginase, TGFbeta, reactive oxygen species).^25^ Interestingly, we found a number of transcripts associated with the reactive oxygen species pathway and oxidative stress among the A36, A37 and A38 modules and for which the expression was highly restricted to erythroid cells. These transcripts included GPX4, PRDX2, GCLC, CYB5R3, ATP6V0C and ISCA1. We also observed a striking increase in A37 transcript abundance in metastatic melanoma and liver transplant recipients under maintenance therapy. Both states are characterized by marked immunosuppression and were categorized in the high abundance profile cluster for A37. Of the 14 other cohorts in this reference collection, only RSV was present in this same cluster. Others have recently described immunosuppression exerted by CECs in patients with late stage cancer: these cells were found to be at least in part responsible for the impaired T‐cell responses observed in this patient population.^27^ Third, the RSV literature provides indications of this virus’ ability to subvert the immune response.^4^ While the possibility of an involvement of CECs in this immune modulation of the response to RSV is novel, the contribution of the hypo‐responsiveness of the neonatal immune system as an underlying factor to progression to severe RSV infection has been clearly outlined.^46^ A key question could thus center on the possible contribution of CECs to the reduced competence of the neonatal immune system. Indeed, results obtained by Elahi et al in animal models indicate that CEC depletion can restore neonatal immune responsiveness and confer resistance to Listeria infection.^26^ More recently, findings reported in a preprint contributed by Shahbaz et al indicated that the suppressive activity of CECs operated in the context of infection by another respiratory virus, SARS‐CoV‐2.^47^ Assuming that CECs may exert such immunosuppressive activity and drive RSV pathogenesis would entail that Ery+ individuals display reduced immune function. Consistently, we found that A37 abundance levels tended to correlate negatively with that of several aggregates associated with lymphocyte responses. A negative trend was notably observed in the case of aggregate A4 that also comprises modules associated with NK cells/cytotoxic responses (*R* = −0.34, *P* < .001). A similar trend was observed for A27 (plasmablasts/antibody‐producing cells; *R* = −0.22, *P* < .001). While such associations are not particularly strong, they are consistent with immunosuppressive role of CECs. They are also in line with results reported in the same preprint contributed by Shahbaz et al describing CEC's suppressive activities in COVID‐19 patients.^47^

Taken together, this work permitted conceptual advances to be made on two fronts. On one hand, we made methodological advances with data integration and meta‐analysis being carried out at the transcriptional module level, rather than at the gene level. It is this data reduction step that permitted the implementation of an original approach to the definition of blood transcriptional “traits” and phenotypes. Furthermore, since the transcriptional module repertoire employed as a framework for these analyses was pre‐established, data interpretation also benefitted from extensive prior functional annotation work and tailored bioinformatic resources.^19^, ^48^ And it is in part thanks to the availability of such resources that potential conceptual advances concerning RSV immunobiology were made on the other hand. Indeed, we previously reported an association between an erythrocyte signature and severe presentation of RSV disease in a subset of the subjects used in the present meta‐analysis.^7^ Here, we confirmed this association in a much larger set of patients. But we were also able to establish links between this A37/Ery+ signature and: (a) immunosuppressive states encountered in melanoma and liver transplant recipients under maintenance therapy; (b) fetal GlyA+ erythroid precursors; and (c) literature attributing immunosuppressive properties of erythroid cells, including in the context of neonatal infections.

Certain limitations to the work presented here must be noted and considered when designing follow‐on studies. First, the hypotheses advanced regarding a possible role of CECs in the context of RSV pathogenesis would need to be validated in patients and animal models using standard immune profiling methods and functional assays. Second, follow‐on studies specifically aimed at determining the potential clinical utility of assessing patient status for the Ery and Infl traits (and potentially others) are needed. Such studies should permit profiling to be conducted in large patient cohorts, using uniform methodologies, and ensure uniform collection of sufficiently detailed clinical data. Unequal depth and lack of uniformity of the clinical information available in public repositories indeed being a clear limitation of meta‐analysis, such as ours presented here. A line of investigation that might be considered for such follow‐on studies would consist of determining utility for predicting risk of developing severe disease in symptomatic patients admitted to the emergency room (as an indication for inpatient care and potential admission to the ICU). Similarly, another line of investigation could focus on assessing the risk of developing severe disease in asymptomatic patients. Information gained from such studies might in turn be used to indicate a need for monoclonal antibody prophylaxis.^49^, ^50^ Such studies might take years to fund and implement; as such, we believe that the publication of early‐stage findings derived from extensive meta‐analysis of large‐scale data is warranted as a first step.

Another limitation is inherent to the profiling of bulk blood RNAseq: while this approach presents the advantage of practicality and scalability, and for these reasons also carries significant translational potential, it does not necessarily allow the delineation of responses from rare cell populations, such as regulatory T cells. Likewise, it may be difficult to distinguish cell subsets, such as myeloid‐derived suppressor cells, whose expression profiles may closely relate to that of other abundant populations. In designing follow‐on studies, it may thus be worthwhile considering to implement, at least in a small subset of patients, single‐cell RNAseq approaches.

In conclusion, the synthesis that we conducted here extends earlier findings, but also offers new avenues of investigations. Notably, it points to the potential clinical relevance of blood transcriptional phenotyping for stratification of patients with RSV infection. More specifically, a signature putatively attributed to immunosuppressive erythroid cells was found to be associated with clinical severity, even in homogenously younger patients. The clinical relevance of this candidate biomarker signature now needs careful assessment. Further investigations into relevance of the CEC population in the context of RSV infection are also warranted. One of the central questions to address is whether these cells merely accompany clinical worsening of the disease or constitute one of its drivers. More generally, this work also highlights the need for follow on, large‐scale blood transcriptome profiling studies of responses to RSV patients, especially over multiple time points and possibly carried out concomitantly in both adult and pediatric populations. Coordination and cooperation between the research groups that might engage in such endeavors would likely prove beneficial for generating large, interoperable blood transcriptome dataset collections.

## 4 METHODS

### 4.1 Selection of public blood transcriptome datasets

Datasets deposited in the NCBI Gene Expression Omnibus (GEO) were used in this meta‐analysis. Accession IDs along with descriptive information and references can be found in Table 1. A reference dataset, which consisted of transcriptome profiles derived from adult blood cell populations and cord blood, was also used to support the functional interpretation of our findings. This dataset was contributed to GEO by Novershtern et al with accession ID GSE24759.^24^

### 4.2 Module repertoire construction

The construction of a transcriptional module repertoire for blood transcriptome analyses has been described previously.^51^, ^52^ The version that was used in this study is the third one developed by our group and is the object of a separate publication (available on a preprint server^19^). Briefly, the approach consists of identifying sets of coexpressed transcripts for a given biological system (in this case blood) and across a wide range of disease or physiological states (perturbations of steady state). In this case, coexpression was determined based on patterns of coclustering observed for all gene pairs across a collection of 16 reference datasets. These datasets encompassed viral and bacterial infectious diseases (HIV, influenza, RSV, melioidosis, *S. aureus*, tuberculosis) as well as several inflammatory or autoimmune diseases (systemic lupus erythematosus, multiple sclerosis, chronic obstructive pulmonary disease, Kawasaki disease, juvenile dermatomyosistis, systemic onset juvenile idiopathic arthritis), B‐cell deficiency, liver transplantation, stage IV melanoma, and pregnancy. The overall collection comprised 985 blood transcriptome profiles. A weighted coexpression network was built based on the coclustering patterns that were obtained. Here, the weight of the nodes connecting a gene pair being based on the number of times coclustering was observed, thus ranging from a weight of one (where coclustering occurs in one of 16 datasets) to a weight of 16 (where coclustering occurs in all 16 datasets). Next, this network was mined using a graph theory algorithm (identification of cliques and paracliques) to define a subset of densely connected gene sets that constituted the module repertoire. Overall, 382 modules were identified via this process, encompassing 14 168 transcripts. A Supporting File including the definition of this module repertoire along with the functional annotations is available from a separate publication.^19^

### 4.3 Constitution of module aggregates

To maintain the number of variables within a manageable number and to facilitate data interpretation, a second tier of clustering was performed to group the modules into “aggregates.” This was achieved by segregating the set of 382 modules according to the patterns of transcript abundance across the 16 reference datasets that were used for module construction. This segregation resulted in the formation of 38 aggregates, each comprising between one and 42 modules. The second level of granularity that was thus obtained was used to define distinct RSV blood transcriptome phenotypes and as a basis for the fingerprint grid plot representation (see Figures 1 and 7). With such grids, the first vertical reading of the fingerprint grid provides an overview of the changes in transcript abundance observed among module aggregates, whereas the horizontal reading provides the changes observed within an aggregate and across modules.

### 4.4 Module repertoire analysis workflow

The modular analyses were performed using the “BloodGen3Module” R package “https://github.com/Drinchai/BloodGen3Module.”^53^ *t*‐test was performed on the log2‐transformed data (FC cutoff = 1.5; FDR cutoff = 0.1). For individual patient analyses, each sample was compared to the mean value of control samples in each dataset. The cutoff comprised an absolute FC > 1.5 and a difference in gene expression level >10. The results for each module analysis are reported as the percentage of its constitutive transcripts for which the abundance was increased or decreased. Because gene sets were selected based on the coexpression observed in blood, the changes in abundance within a given module tended to be coordinated and the dominant trend selected (the greater value of the percentage increased vs percentage decreased). A module was considered to be “responsive” when the proportion of differentially expressed transcripts (as defined above) was >15%.

### 4.5 Data visualization

The results were visualized in a fingerprint format, either as a grid plot (group level, Figure 1) or as a heat map (individual level, Figure 5), using the same illustrative RSV dataset. For each module, the percentage of increased transcripts is represented by a red spot and the percentage of decreased transcripts is represented by a blue spot. The larger of the two values was retained for visualization. In the grid format (Figure 1), the position of each module is fixed. A row of modules corresponds to a “module aggregate,” which as described above is a set of modules following a similar pattern of activity across the 16 input datasets corresponding to different disease or physiological states. A few “aggregates” comprised only a single module and thus are not shown on the grid.

For the heat maps (Figures 2, 5, and 7B), each row corresponds to a module and each column to a sample. The columns and rows are arranged based on similarities in the patterns of module activity. Filters can be applied to remove modules that show only low levels of activity across the samples or to retain only the modules associated with functional annotations.

The fingerprint grid plots and heat maps were generated using the “BloodGen3Module” R package (link provided above)”^53^

## FUNDING INFORMATION

AP‐HP, Pitié‐Salpêtrière Hospital, LabEx Transimmunom, Grant Number: ANR‐11‐IDEX‐0004‐02; RHU iMAP; Open Access funding provided by the Qatar National Library.

## AUTHOR CONTRIBUTIONS

Conceptualization: Darawan Rinchai, Damien Chaussabel, Davide Bedognetti, Encarnita Mariotti‐Ferrandiz, David Klatzmann, Karolina Palucka, and Matthew B. Altman. Data curation and validation: Darawan Rinchai, Mohammed Toufiq, Mathieu Garand, Basirudeen Kabeer, Oceane Konza, Signe Hässler, and Federica Martina. Visualization: Darawan Rinchai, Oceane Konza, Signe Hässler, Federica Martina, and Encarnita Mariotti‐Ferrandiz. Analysis and interpretation: Darawan Rinchai, Oceane Konza, Signe Hässler, Federica Martina, Asuncion Mejias, Octavio Ramilo, Encarnita Mariotti‐Ferrandiz, David Klatzmann, and Damien Chaussabel. Writing of the first draft: Darawan Rinchai and Damien Chaussabel. Funding acquisition: David Klatzmann and Damien Chaussabel. Methodology development: Darawan Rinchai and Damien Chaussabel. Writing, review, and editing: Darawan Rinchai, Matthew B. Altman, Mohammed Toufiq, Mathieu Garand, Basirudeen Kabeer, Oceane Konza, Signe Hässler, Federica Martina, Asuncion Mejias, Octavio Ramilo, Davide Bedognetti, Encarnita Mariotti‐Ferrandiz, David Klatzmann, Karolina Palucka, and Damien Chaussabel. The contributor's roles listed above follow the Contributor Roles Taxonomy (CRediT) managed by The Consortia Advancing Standards in Research Administration Information (CASRAI) (https://casrai.org/credit/).

## CONFLICT OF INTEREST

The authors declare that there is no conflict of interest.

## Supporting information

Supporting Information

Supporting Information

## Data Availability

The data that support the findings of this study are openly available in the NCBI Gene Expression Omnibus at https://www.ncbi.nlm.nih.gov/geo/, with reference numbers provided throughout the text where appropriate.
