# Supplementary material for: Definition of erythroid cell‐positive blood transcriptome phenotypes associated with severe respiratory syncytial virus infection
Source: Clin Transl Med. 2020 Dec 12;10(8):e244. doi: 10.1002/ctm2.244 (PMC7733317; doi:10.1002/ctm2.244)
Supplement: Supplementary file 1 — Supporting Information [file CTM2-10-e244-s001.pdf]

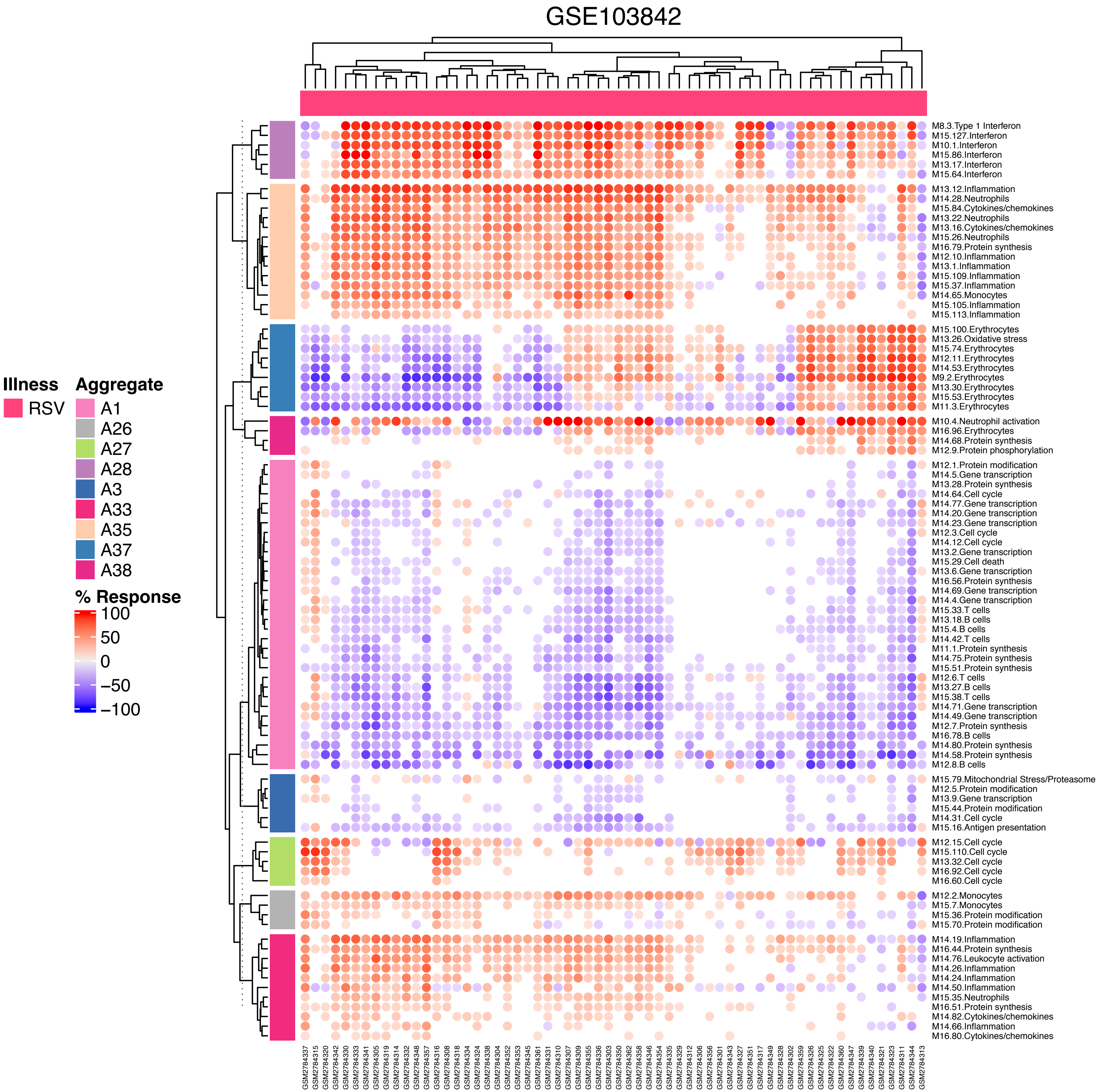
GSE103842
Illness
RSV
Aggregate
A1
A26
A27
A28
A3
A33
A35
A37
A38
% Response
100
50
0
−50
−100
M8.3.Type 1 Interferon
M15.127.Interferon
M10.1.Interferon
M15.86.Interferon
M13.17.Interferon
M15.64.Interferon
M13.12.Inflammation
M14.28.Neutrophils
M15.84.Cytokines/chemokines
M13.22.Neutrophils
M13.16.Cytokines/chemokines
M15.26.Neutrophils
M16.79.Protein synthesis
M12.10.Inflammation
M13.1.Inflammation
M15.109.Inflammation
M15.37.Inflammation
M14.65.Monocytes
M15.105.Inflammation
M15.113.Inflammation
M15.100.Erythrocytes
M13.26.Oxidative stress
M15.74.Erythrocytes
M12.11.Erythrocytes
M14.53.Erythrocytes
M9.2.Erythrocytes
M13.30.Erythrocytes
M15.53.Erythrocytes
M11.3.Erythrocytes
M10.4.Neutrophil activation
M16.96.Erythrocytes
M14.68.Protein synthesis
M12.9.Protein phosphorylation
M12.1.Protein modification
M14.5.Gene transcription
M13.28.Protein synthesis
M14.64.Cell cycle
M14.77.Gene transcription
M14.20.Gene transcription
M14.23.Gene transcription
M12.3.Cell cycle
M14.12.Cell cycle
M13.2.Gene transcription
M15.29.Cell death
M13.6.Gene transcription
M16.56.Protein synthesis
M14.69.Gene transcription
M14.4.Gene transcription
M15.33.T cells
M13.18.B cells
M15.4.B cells
M14.42.T cells
M11.1.Protein synthesis
M14.75.Protein synthesis
M15.51.Protein synthesis
M12.6.T cells
M13.27.B cells
M15.38.T cells
M14.71.Gene transcription
M14.49.Gene transcription
M12.7.Protein synthesis
M16.78.B cells
M14.80.Protein synthesis
M14.58.Protein synthesis
M12.8.B cells
M15.79.Mitochondrial Stress/Proteasome
M12.5.Protein modification
M13.9.Gene transcription
M15.44.Protein modification
M14.31.Cell cycle
M15.16.Antigen presentation
M12.15.Cell cycle
M15.110.Cell cycle
M13.32.Cell cycle
M16.92.Cell cycle
M16.60.Cell cycle
M12.2.Monocytes
M15.7.Monocytes
M15.36.Protein modification
M15.70.Protein modification
M14.19.Inflammation
M16.44.Protein synthesis
M14.76.Leukocyte activation
M14.26.Inflammation
M14.24.Inflammation
M14.50.Inflammation
M15.35.Neutrophils
M16.51.Protein synthesis
M14.82.Cytokines/chemokines
M14.66.Inflammation
M16.80.Cytokines/chemokines

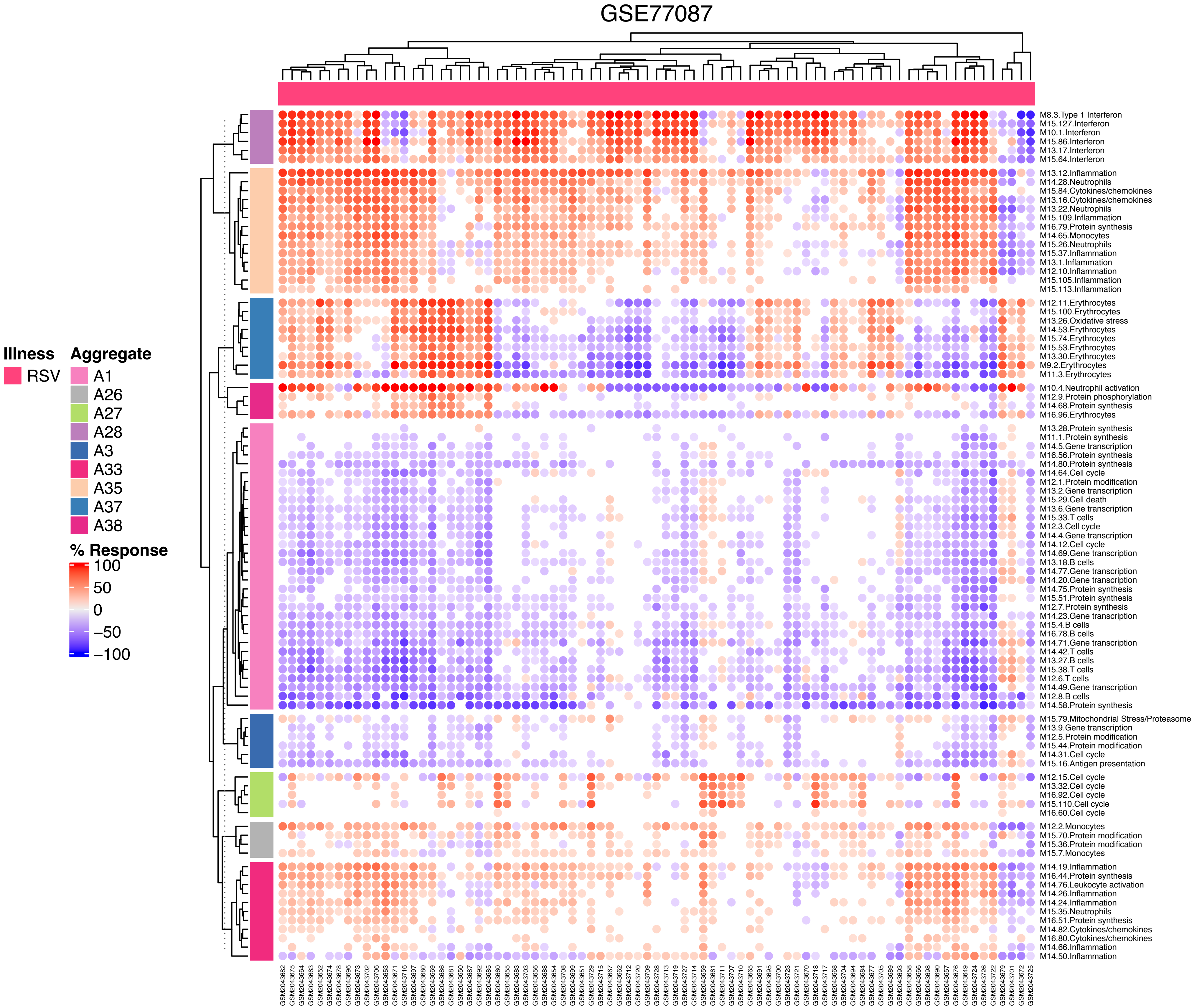
GSE77087
Illness
RSV
Aggregate
A1
A26
A27
A28
A3
A33
A35
A37
A38
% Response
100
50
0
−50
−100
M8.3.Type 1 Interferon
M15.127.Interferon
M10.1.Interferon
M15.86.Interferon
M13.17.Interferon
M15.64.Interferon
M13.12.Inflammation
M14.28.Neutrophils
M15.84.Cytokines/chemokines
M13.16.Cytokines/chemokines
M13.22.Neutrophils
M15.109.Inflammation
M16.79.Protein synthesis
M14.65.Monocytes
M15.26.Neutrophils
M15.37.Inflammation
M13.1.Inflammation
M12.10.Inflammation
M15.105.Inflammation
M15.113.Inflammation
M12.11.Erythrocytes
M15.100.Erythrocytes
M13.26.Oxidative stress
M14.53.Erythrocytes
M15.74.Erythrocytes
M15.53.Erythrocytes
M13.30.Erythrocytes
M9.2.Erythrocytes
M11.3.Erythrocytes
M10.4.Neutrophil activation
M12.9.Protein phosphorylation
M14.68.Protein synthesis
M16.96.Erythrocytes
M13.28.Protein synthesis
M11.1.Protein synthesis
M14.5.Gene transcription
M16.56.Protein synthesis
M14.80.Protein synthesis
M14.64.Cell cycle
M12.1.Protein modification
M13.2.Gene transcription
M15.29.Cell death
M13.6.Gene transcription
M15.33.T cells
M12.3.Cell cycle
M14.4.Gene transcription
M14.12.Cell cycle
M14.69.Gene transcription
M13.18.B cells
M14.77.Gene transcription
M14.20.Gene transcription
M14.75.Protein synthesis
M15.51.Protein synthesis
M12.7.Protein synthesis
M14.23.Gene transcription
M15.4.B cells
M16.78.B cells
M14.71.Gene transcription
M14.42.T cells
M13.27.B cells
M15.38.T cells
M12.6.T cells
M14.49.Gene transcription
M12.8.B cells
M14.58.Protein synthesis
M15.79.Mitochondrial Stress/Proteasome
M13.9.Gene transcription
M12.5.Protein modification
M15.44.Protein modification
M14.31.Cell cycle
M15.16.Antigen presentation
M12.15.Cell cycle
M13.32.Cell cycle
M16.92.Cell cycle
M15.110.Cell cycle
M16.60.Cell cycle
M12.2.Monocytes
M15.70.Protein modification
M15.36.Protein modification
M15.7.Monocytes
M14.19.Inflammation
M16.44.Protein synthesis
M14.76.Leukocyte activation
M14.26.Inflammation
M14.24.Inflammation
M15.35.Neutrophils
M16.51.Protein synthesis
M14.82.Cytokines/chemokines
M16.80.Cytokines/chemokines
M14.66.Inflammation
M14.50.Inflammation

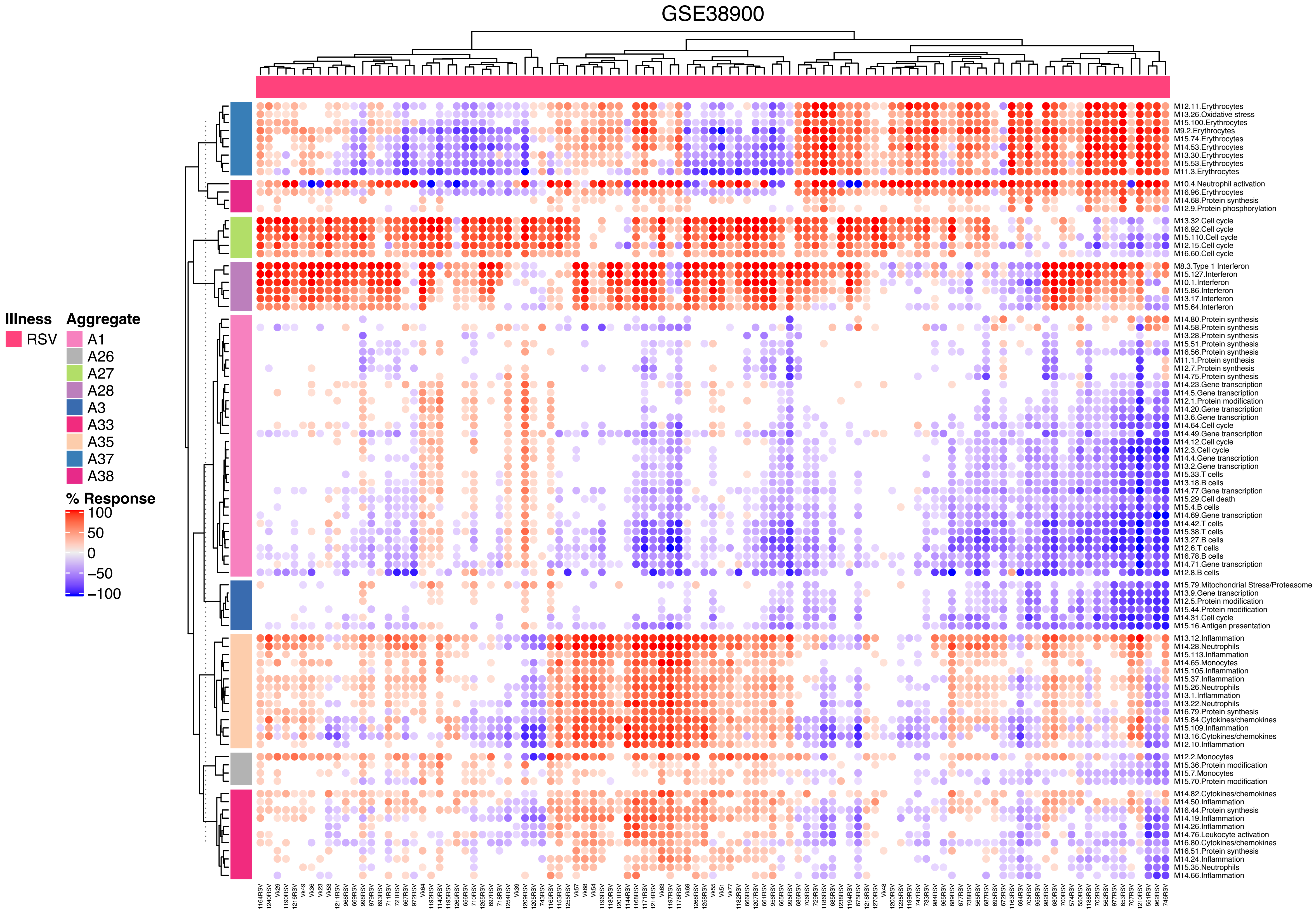
GSE38900
Illness
RSV
Aggregate
A1
A26
A27
A28
A3
A33
A35
A37
A38
% Response
100
50
0
−50
−100
M12.11.Erythrocytes
M13.26.Oxidative stress
M15.100.Erythrocytes
M9.2.Erythrocytes
M15.74.Erythrocytes
M14.53.Erythrocytes
M13.30.Erythrocytes
M15.53.Erythrocytes
M11.3.Erythrocytes
M10.4.Neutrophil activation
M16.96.Erythrocytes
M14.68.Protein synthesis
M12.9.Protein phosphorylation
M13.32.Cell cycle
M16.92.Cell cycle
M15.110.Cell cycle
M12.15.Cell cycle
M16.60.Cell cycle
M8.3.Type 1 Interferon
M15.127.Interferon
M10.1.Interferon
M15.86.Interferon
M13.17.Interferon
M15.64.Interferon
M14.80.Protein synthesis
M14.58.Protein synthesis
M13.28.Protein synthesis
M15.51.Protein synthesis
M16.56.Protein synthesis
M11.1.Protein synthesis
M12.7.Protein synthesis
M14.75.Protein synthesis
M14.23.Gene transcription
M14.5.Gene transcription
M12.1.Protein modification
M14.20.Gene transcription
M13.6.Gene transcription
M14.64.Cell cycle
M14.49.Gene transcription
M14.12.Cell cycle
M12.3.Cell cycle
M14.4.Gene transcription
M13.2.Gene transcription
M15.33.T cells
M13.18.B cells
M14.77.Gene transcription
M15.29.Cell death
M15.4.B cells
M14.69.Gene transcription
M14.42.T cells
M15.38.T cells
M13.27.B cells
M12.6.T cells
M16.78.B cells
M14.71.Gene transcription
M12.8.B cells
M15.79.Mitochondrial Stress/Proteasome
M13.9.Gene transcription
M12.5.Protein modification
M15.44.Protein modification
M14.31.Cell cycle
M15.16.Antigen presentation
M13.12.Inflammation
M14.28.Neutrophils
M15.113.Inflammation
M14.65.Monocytes
M15.105.Inflammation
M15.37.Inflammation
M15.26.Neutrophils
M13.1.Inflammation
M13.22.Neutrophils
M16.79.Protein synthesis
M15.84.Cytokines/chemokines
M15.109.Inflammation
M13.16.Cytokines/chemokines
M12.10.Inflammation
M12.2.Monocytes
M15.36.Protein modification
M15.7.Monocytes
M15.70.Protein modification
M14.82.Cytokines/chemokines
M14.50.Inflammation
M16.44.Protein synthesis
M14.19.Inflammation
M14.26.Inflammation
M14.76.Leukocyte activation
M16.80.Cytokines/chemokines
M16.51.Protein synthesis
M14.24.Inflammation
M15.35.Neutrophils
M14.66.Inflammation

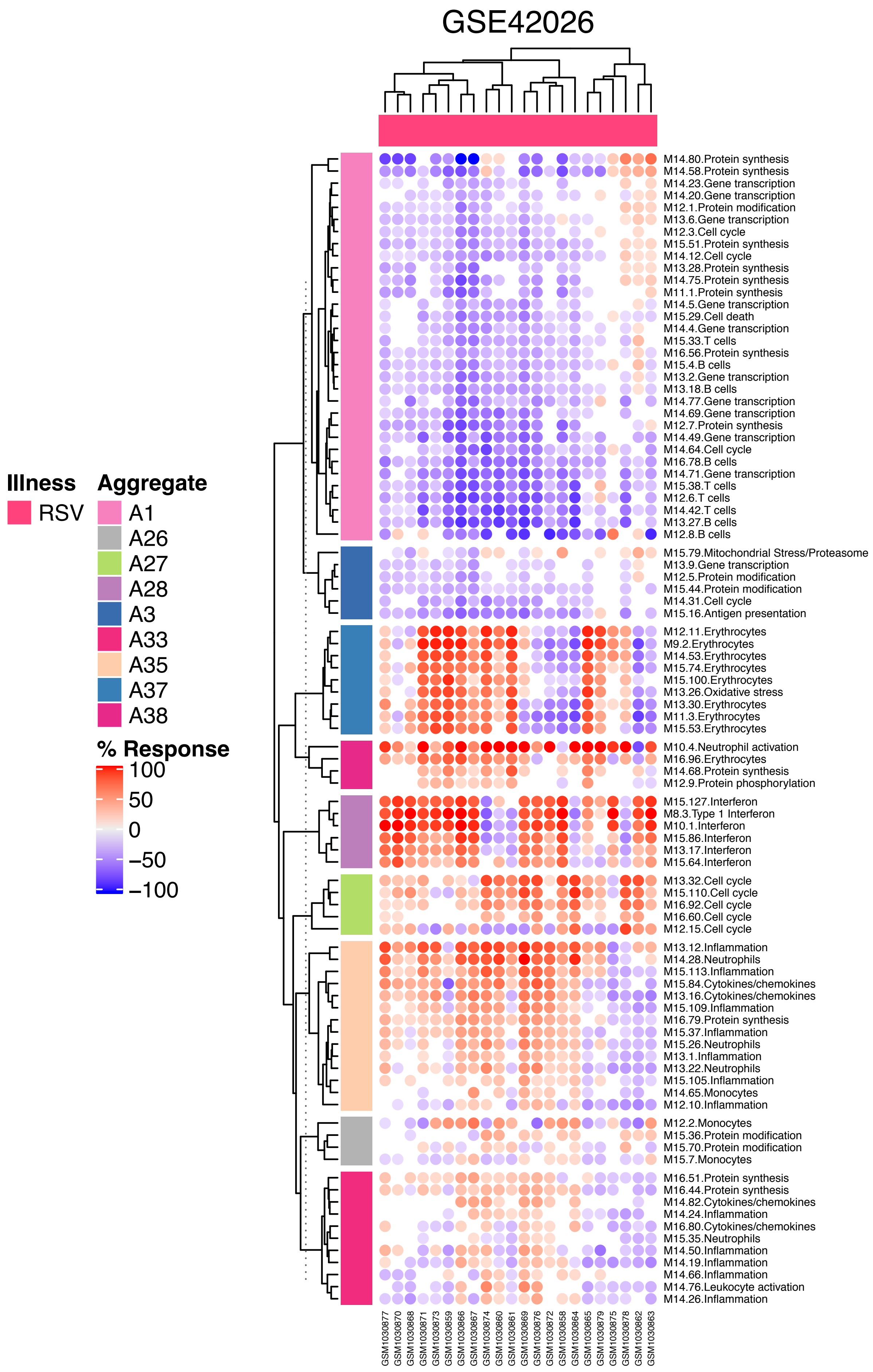
GSE42026
Illness
RSV
Aggregate
A1
A26
A27
A28
A3
A33
A35
A37
A38
% Response
100
50
0
−50
−100
M14.80.Protein synthesis
M14.58.Protein synthesis
M14.23.Gene transcription
M14.20.Gene transcription
M12.1.Protein modification
M13.6.Gene transcription
M12.3.Cell cycle
M15.51.Protein synthesis
M14.12.Cell cycle
M13.28.Protein synthesis
M14.75.Protein synthesis
M11.1.Protein synthesis
M14.5.Gene transcription
M15.29.Cell death
M14.4.Gene transcription
M15.33.T cells
M16.56.Protein synthesis
M15.4.B cells
M13.2.Gene transcription
M13.18.B cells
M14.77.Gene transcription
M14.69.Gene transcription
M12.7.Protein synthesis
M14.49.Gene transcription
M14.64.Cell cycle
M16.78.B cells
M14.71.Gene transcription
M15.38.T cells
M12.6.T cells
M14.42.T cells
M13.27.B cells
M12.8.B cells
M15.79.Mitochondrial Stress/Proteasome
M13.9.Gene transcription
M12.5.Protein modification
M15.44.Protein modification
M14.31.Cell cycle
M15.16.Antigen presentation
M12.11.Erythrocytes
M9.2.Erythrocytes
M14.53.Erythrocytes
M15.74.Erythrocytes
M15.100.Erythrocytes
M13.26.Oxidative stress
M13.30.Erythrocytes
M11.3.Erythrocytes
M15.53.Erythrocytes
M10.4.Neutrophil activation
M16.96.Erythrocytes
M14.68.Protein synthesis
M12.9.Protein phosphorylation
M15.127.Interferon
M8.3.Type 1 Interferon
M10.1.Interferon
M15.86.Interferon
M13.17.Interferon
M15.64.Interferon
M13.32.Cell cycle
M15.110.Cell cycle
M16.92.Cell cycle
M16.60.Cell cycle
M12.15.Cell cycle
M13.12.Inflammation
M14.28.Neutrophils
M15.113.Inflammation
M15.84.Cytokines/chemokines
M13.16.Cytokines/chemokines
M15.109.Inflammation
M16.79.Protein synthesis
M15.37.Inflammation
M15.26.Neutrophils
M13.1.Inflammation
M13.22.Neutrophils
M15.105.Inflammation
M14.65.Monocytes
M12.10.Inflammation
M12.2.Monocytes
M15.36.Protein modification
M15.70.Protein modification
M15.7.Monocytes
M16.51.Protein synthesis
M16.44.Protein synthesis
M14.82.Cytokines/chemokines
M14.24.Inflammation
M16.80.Cytokines/chemokines
M15.35.Neutrophils
M14.50.Inflammation
M14.19.Inflammation
M14.66.Inflammation
M14.76.Leukocyte activation
M14.26.Inflammation

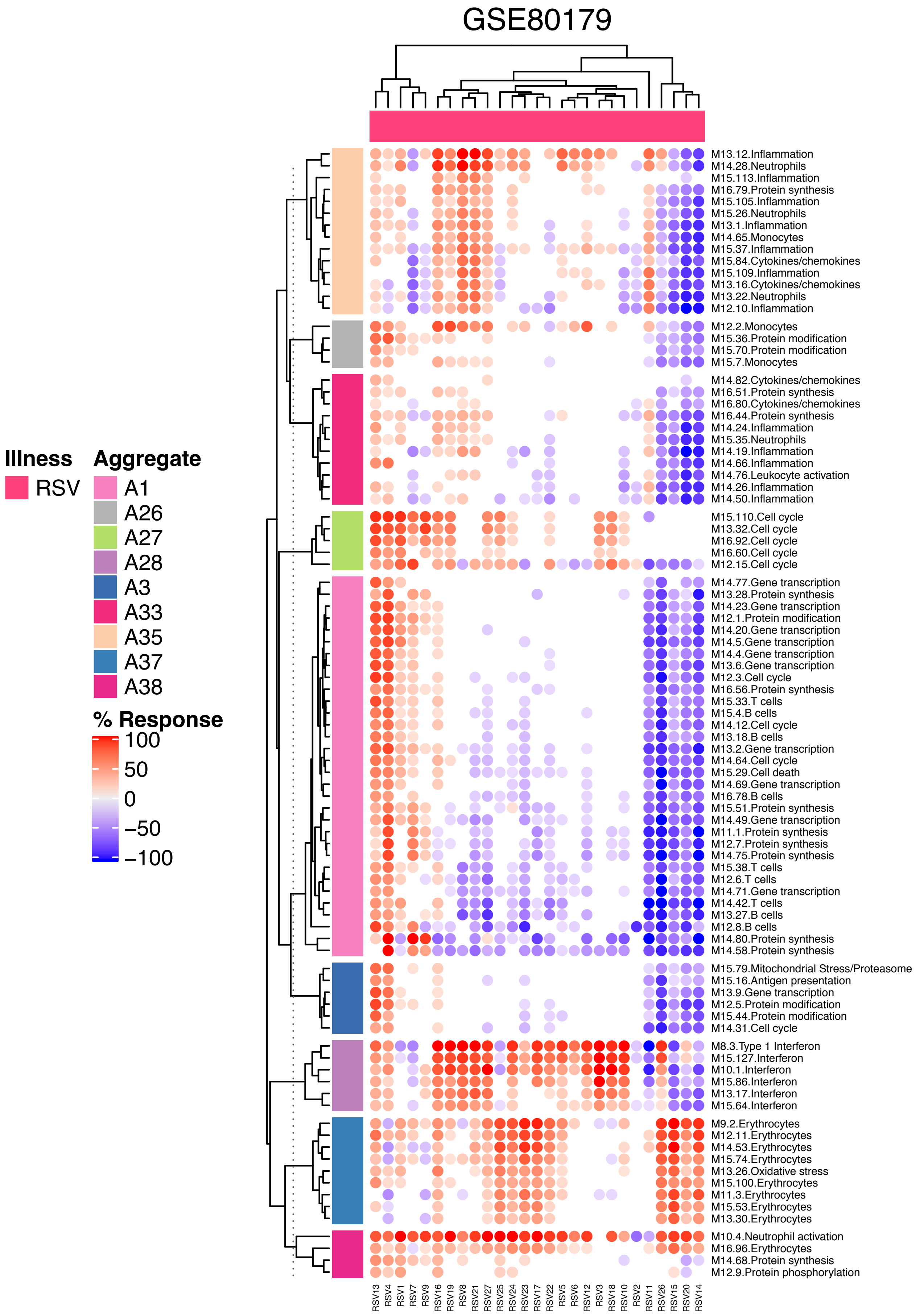
GSE80179
Illness
RSV
Aggregate
A1
A26
A27
A28
A3
A33
A35
A37
A38
% Response
100
50
0
−50
−100
M13.12.Inflammation
M14.28.Neutrophils
M15.113.Inflammation
M16.79.Protein synthesis
M15.105.Inflammation
M15.26.Neutrophils
M13.1.Inflammation
M14.65.Monocytes
M15.37.Inflammation
M15.84.Cytokines/chemokines
M15.109.Inflammation
M13.16.Cytokines/chemokines
M13.22.Neutrophils
M12.10.Inflammation
M12.2.Monocytes
M15.36.Protein modification
M15.70.Protein modification
M15.7.Monocytes
M14.82.Cytokines/chemokines
M16.51.Protein synthesis
M16.80.Cytokines/chemokines
M16.44.Protein synthesis
M14.24.Inflammation
M15.35.Neutrophils
M14.19.Inflammation
M14.66.Inflammation
M14.76.Leukocyte activation
M14.26.Inflammation
M14.50.Inflammation
M15.110.Cell cycle
M13.32.Cell cycle
M16.92.Cell cycle
M16.60.Cell cycle
M12.15.Cell cycle
M14.77.Gene transcription
M13.28.Protein synthesis
M14.23.Gene transcription
M12.1.Protein modification
M14.20.Gene transcription
M14.5.Gene transcription
M14.4.Gene transcription
M13.6.Gene transcription
M12.3.Cell cycle
M16.56.Protein synthesis
M15.33.T cells
M15.4.B cells
M14.12.Cell cycle
M13.18.B cells
M13.2.Gene transcription
M14.64.Cell cycle
M15.29.Cell death
M14.69.Gene transcription
M16.78.B cells
M15.51.Protein synthesis
M14.49.Gene transcription
M11.1.Protein synthesis
M12.7.Protein synthesis
M14.75.Protein synthesis
M15.38.T cells
M12.6.T cells
M14.71.Gene transcription
M14.42.T cells
M13.27.B cells
M12.8.B cells
M14.80.Protein synthesis
M14.58.Protein synthesis
M15.79.Mitochondrial Stress/Proteasome
M15.16.Antigen presentation
M13.9.Gene transcription
M12.5.Protein modification
M15.44.Protein modification
M14.31.Cell cycle
M8.3.Type 1 Interferon
M15.127.Interferon
M10.1.Interferon
M15.86.Interferon
M13.17.Interferon
M15.64.Interferon
M9.2.Erythrocytes
M12.11.Erythrocytes
M14.53.Erythrocytes
M15.74.Erythrocytes
M13.26.Oxidative stress
M15.100.Erythrocytes
M11.3.Erythrocytes
M15.53.Erythrocytes
M13.30.Erythrocytes
M10.4.Neutrophil activation
M16.96.Erythrocytes
M14.68.Protein synthesis
M12.9.Protein phosphorylation
RSV13
RSV4
RSV1
RSV7
RSV9
RSV16
RSV19
RSV8
RSV21
RSV27
RSV25
RSV24
RSV23
RSV17
RSV22
RSV5
RSV6
RSV12
RSV3
RSV18
RSV10
RSV2
RSV11
RSV26
RSV15
RSV20
RSV14

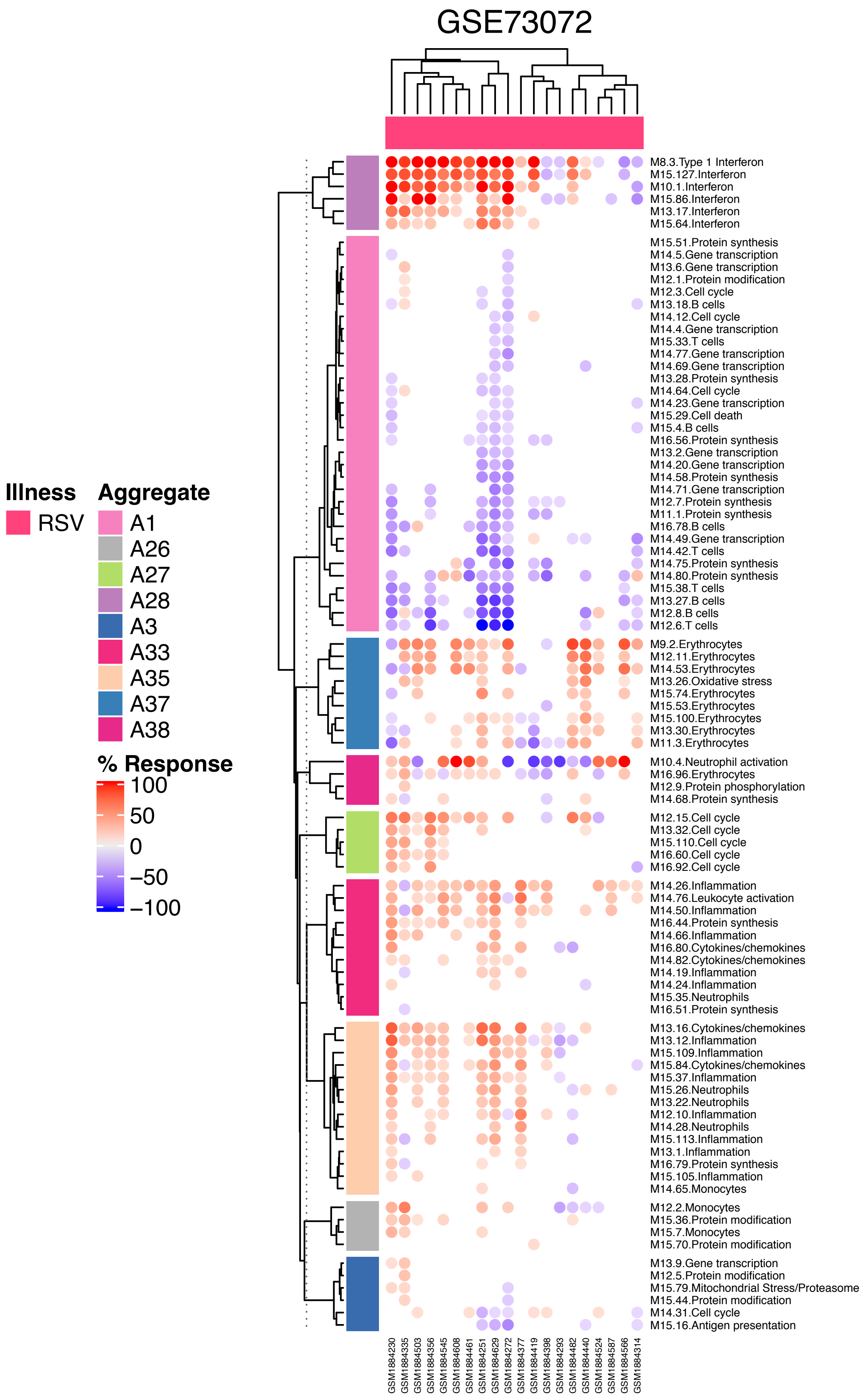
GSE73072
Illness
RSV
Aggregate
A1
A26
A27
A28
A3
A33
A35
A37
A38
% Response
100
50
0
−50
−100
M8.3.Type 1 Interferon
M15.127.Interferon
M10.1.Interferon
M15.86.Interferon
M13.17.Interferon
M15.64.Interferon
M15.51.Protein synthesis
M14.5.Gene transcription
M13.6.Gene transcription
M12.1.Protein modification
M12.3.Cell cycle
M13.18.B cells
M14.12.Cell cycle
M14.4.Gene transcription
M15.33.T cells
M14.77.Gene transcription
M14.69.Gene transcription
M13.28.Protein synthesis
M14.64.Cell cycle
M14.23.Gene transcription
M15.29.Cell death
M15.4.B cells
M16.56.Protein synthesis
M13.2.Gene transcription
M14.20.Gene transcription
M14.58.Protein synthesis
M14.71.Gene transcription
M12.7.Protein synthesis
M11.1.Protein synthesis
M16.78.B cells
M14.49.Gene transcription
M14.42.T cells
M14.75.Protein synthesis
M14.80.Protein synthesis
M15.38.T cells
M13.27.B cells
M12.8.B cells
M12.6.T cells
M9.2.Erythrocytes
M12.11.Erythrocytes
M14.53.Erythrocytes
M13.26.Oxidative stress
M15.74.Erythrocytes
M15.53.Erythrocytes
M15.100.Erythrocytes
M13.30.Erythrocytes
M11.3.Erythrocytes
M10.4.Neutrophil activation
M16.96.Erythrocytes
M12.9.Protein phosphorylation
M14.68.Protein synthesis
M12.15.Cell cycle
M13.32.Cell cycle
M15.110.Cell cycle
M16.60.Cell cycle
M16.92.Cell cycle
M14.26.Inflammation
M14.76.Leukocyte activation
M14.50.Inflammation
M16.44.Protein synthesis
M14.66.Inflammation
M16.80.Cytokines/chemokines
M14.82.Cytokines/chemokines
M14.19.Inflammation
M14.24.Inflammation
M15.35.Neutrophils
M16.51.Protein synthesis
M13.16.Cytokines/chemokines
M13.12.Inflammation
M15.109.Inflammation
M15.84.Cytokines/chemokines
M15.37.Inflammation
M15.26.Neutrophils
M13.22.Neutrophils
M12.10.Inflammation
M14.28.Neutrophils
M15.113.Inflammation
M13.1.Inflammation
M16.79.Protein synthesis
M15.105.Inflammation
M14.65.Monocytes
M12.2.Monocytes
M15.36.Protein modification
M15.7.Monocytes
M15.70.Protein modification
M13.9.Gene transcription
M12.5.Protein modification
M15.79.Mitochondrial Stress/Proteasome
M15.44.Protein modification
M14.31.Cell cycle
M15.16.Antigen presentation
GSM1884230
GSM1884335
GSM1884503
GSM1884356
GSM1884545
GSM1884608
GSM1884461
GSM1884251
GSM1884629
GSM1884272
GSM1884377
GSM1884419
GSM1884398
GSM1884293
GSM1884482
GSM1884440
GSM1884524
GSM1884587
GSM1884566
GSM1884314
