# Supplementary material for: Definition of erythroid cell‐positive blood transcriptome phenotypes associated with severe respiratory syncytial virus infection
Source: Clin Transl Med. 2020 Dec 12;10(8):e244. doi: 10.1002/ctm2.244 (PMC7733317; doi:10.1002/ctm2.244)
Supplement: Supplementary file 2 — Supporting Information [file CTM2-10-e244-s002.pdf]

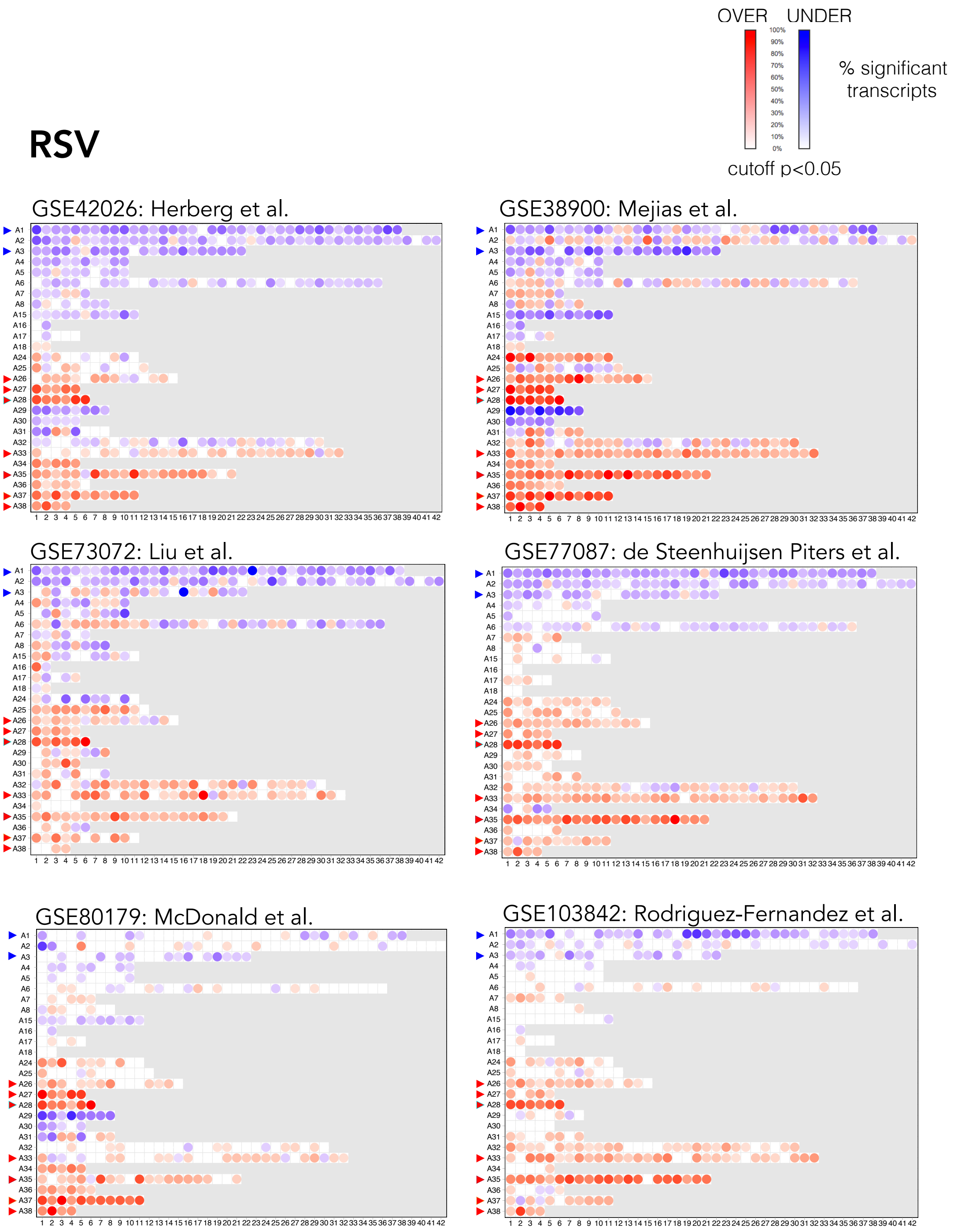

Supplementary Figure 1: Fingerprint grid maps of modular repertoire changes in six independent RSV blood transcriptome datasets. The visualization scheme is similar to the one described for the fingerprint grid map in Figure 1B. Because the position of the modules on the grid is fixed, the color key from Figure 1 can also be used for functional interpretation. The maps were generated from multiple independent RSV blood transcriptome datasets that are available in the NCBI Gene Expression Omnibus (5, 6, 20–22, 28).

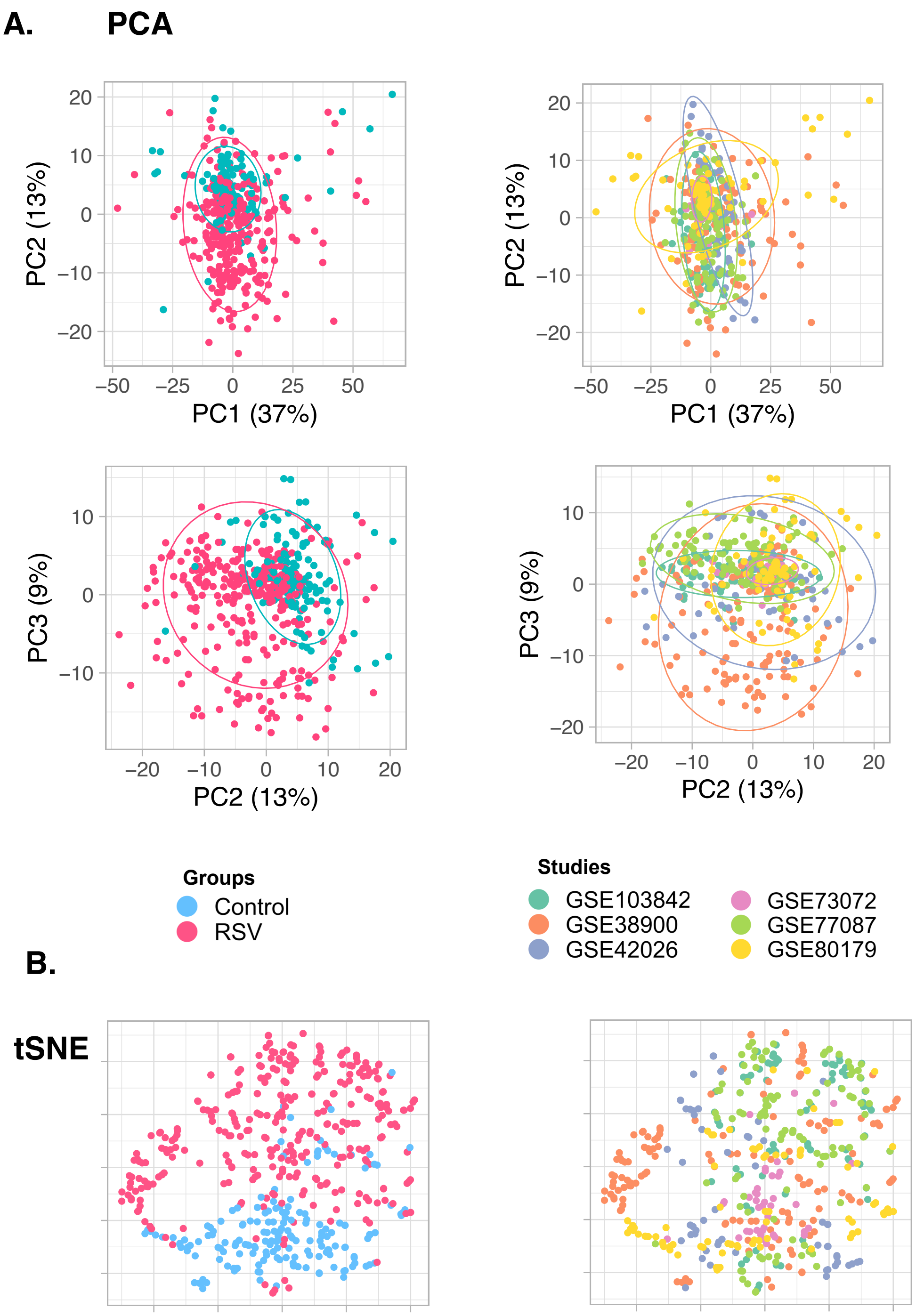

Supplementary Figure 2: Individual subjects PCA plots and tSNE plots. The modular fingerprint profiles of individual subjects from each of the six different studies were combined and PCA and tSNE plots were generated. The total number of subjects was 490 (319 subjects with RSV infection and 171 controls). A. 2D PCA plots. The subjects are color-coded according to grouping information (Groups) and dataset membership (Studies). B. tSNE plots. In both types of plot, each sample is represented by a dot. The proximity between the dots is an indication of the modular transcriptional profile similarity.

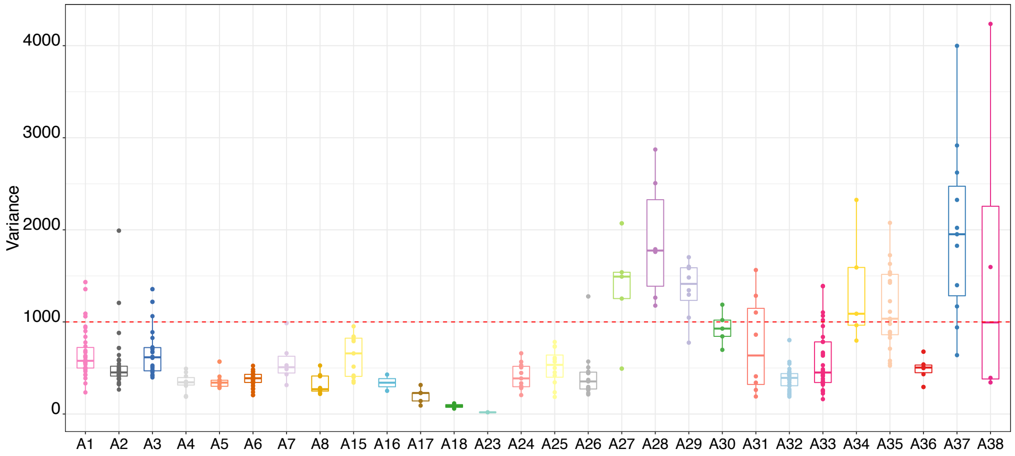

Supplementary Figure 3: Inter-individual variance of summarized module-level abundance values. The variance in the percentage of the transcript response across 319 RSV patients was calculated across 382 modules. The box plots display those values with each module being grouped in its respective module aggregate, from A1 to A38.

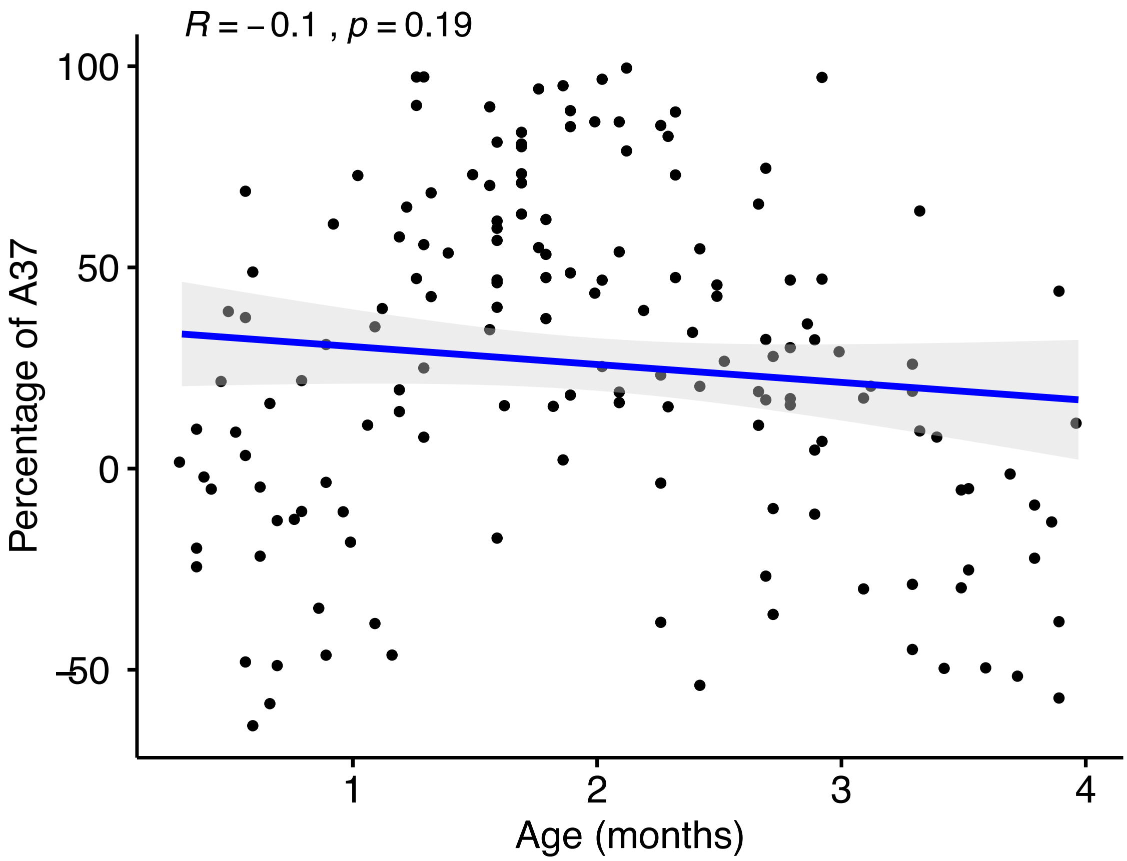

Supplementary Figure 4: Association between aggregate A37 abundance levels and the age for subject less than 4 months old. This correlation plot represents levels of abundance of transcripts constituting aggregate A37 (Erythroid cells) and the age of a patients comprised in our consolidated cohort who are less than 4 months old. Transcript abundance is expressed as a percentage of transcripts within modules constituting this aggregate that meet our significance cutoff (e.g. -100% means abundance of all genes within these modules are decreased compared to uninfected controls; +100% means that abundance of all genes within these modules are decreased).

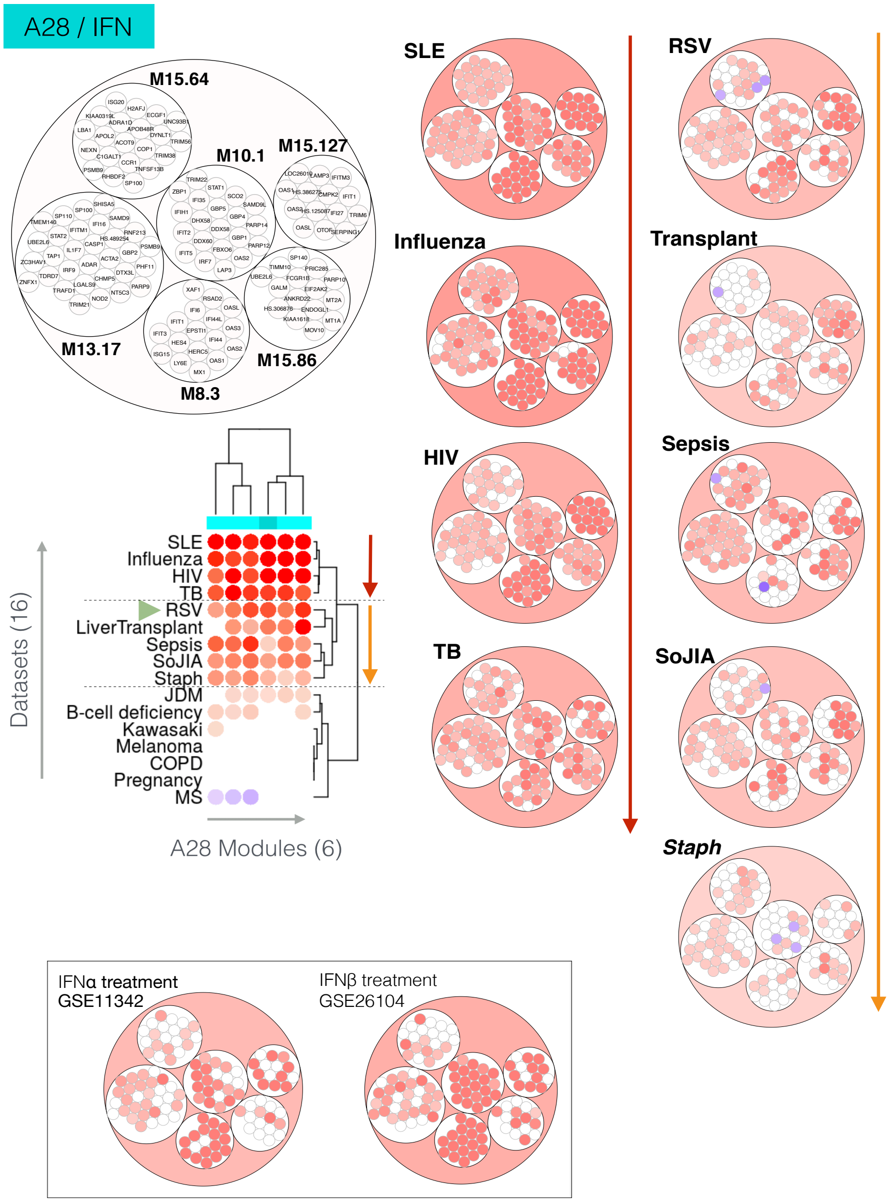

Supplementary Figure 5: Patterns of abundance of six A28 modules across several disease and physiological states. Each column on the heatmap corresponds to one of six modules constituting the A28 aggregate. Each row corresponds to one of 16 reference datasets. Red spots on the heatmap indicate an increase in abundance of the transcripts constituting a given module for a given dataset. A blue spot indicates a decrease in abundance. No color indicates no change. Disease or physiological states were arranged based on similarity in patterns of aggregate activity. The circle (top left) is a representation of the six modules constituting aggregate 28, and of the transcripts constituting each of the modules. Some genes on the Illumina BeadArrays can map to multiple probes, which explains the few instances where the same gene can be found in different modules. The smaller circles (right) represent changes in transcript abundance of individual transcripts for two of the disease clusters. The first cluster (red arrow) comprises four diseases with the highest degree of increase in transcript abundance. The second cluster (orange arrow) comprises five diseases with an intermediate level of increase in abundance. The insert (bottom) shows two circles representing changes in abundance in patients with hepatitis C treated with IFNα (48), and patients with multiple sclerosis treated with IFNβ (49). The corresponding NCBI GEO accession IDs are indicated for each.

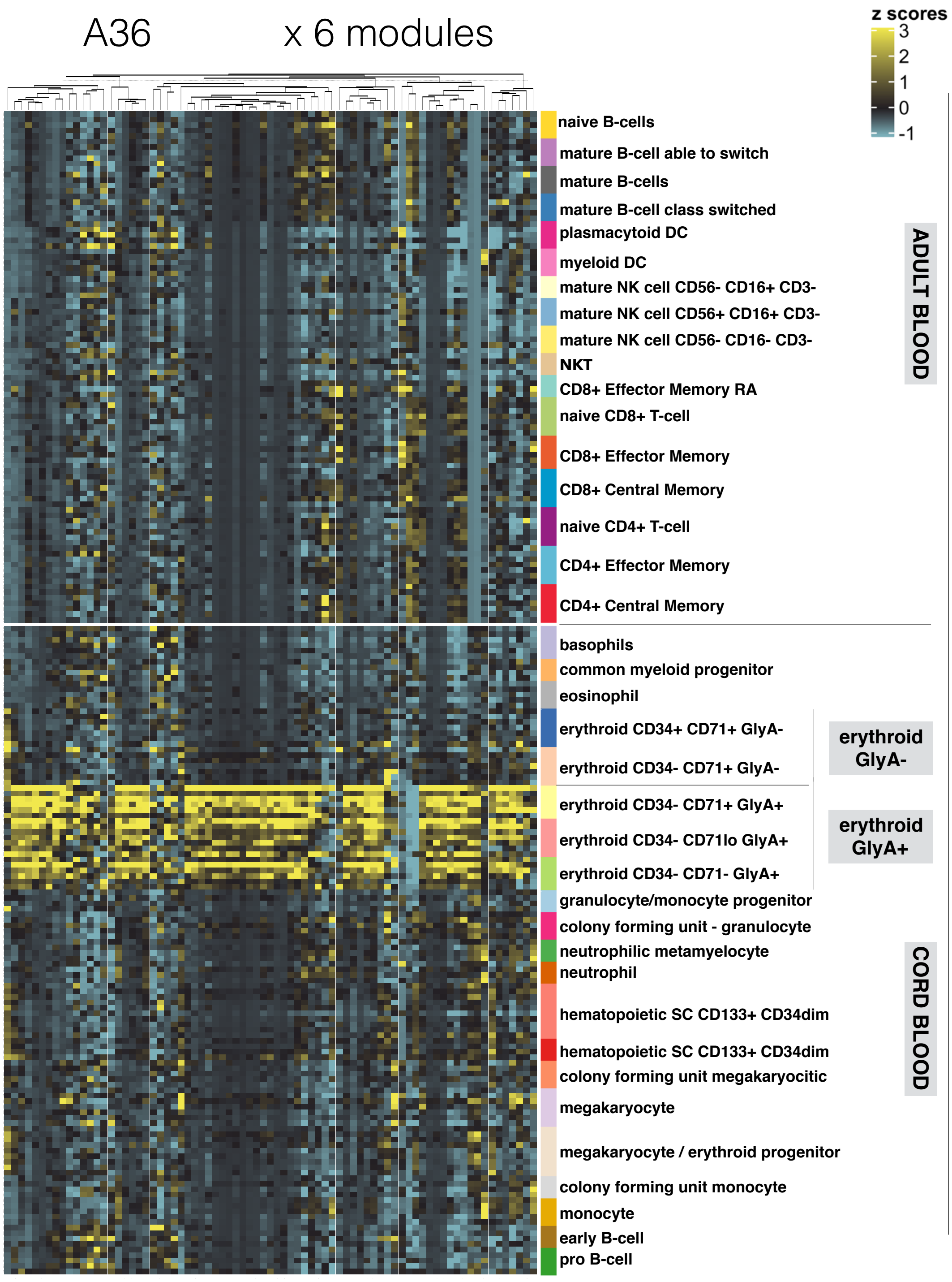

Supplementary Figure 6: The expression levels of A36 genes across the cell populations isolated from human peripheral blood and cord blood. The abundance levels of transcripts comprised in the 11 modules constituting aggregate A36 (columns) across blood cell populations (rows). The dataset is publicly available under GEO accession ID GSE24759 (24). The populations are separated based on whether they were isolated from adult venous blood (top) or from neonate cord blood (bottom). Distinct erythroid cell populations isolated on the basis of cell surface expression of CD34, CD71 and GlyA antigens are shown.

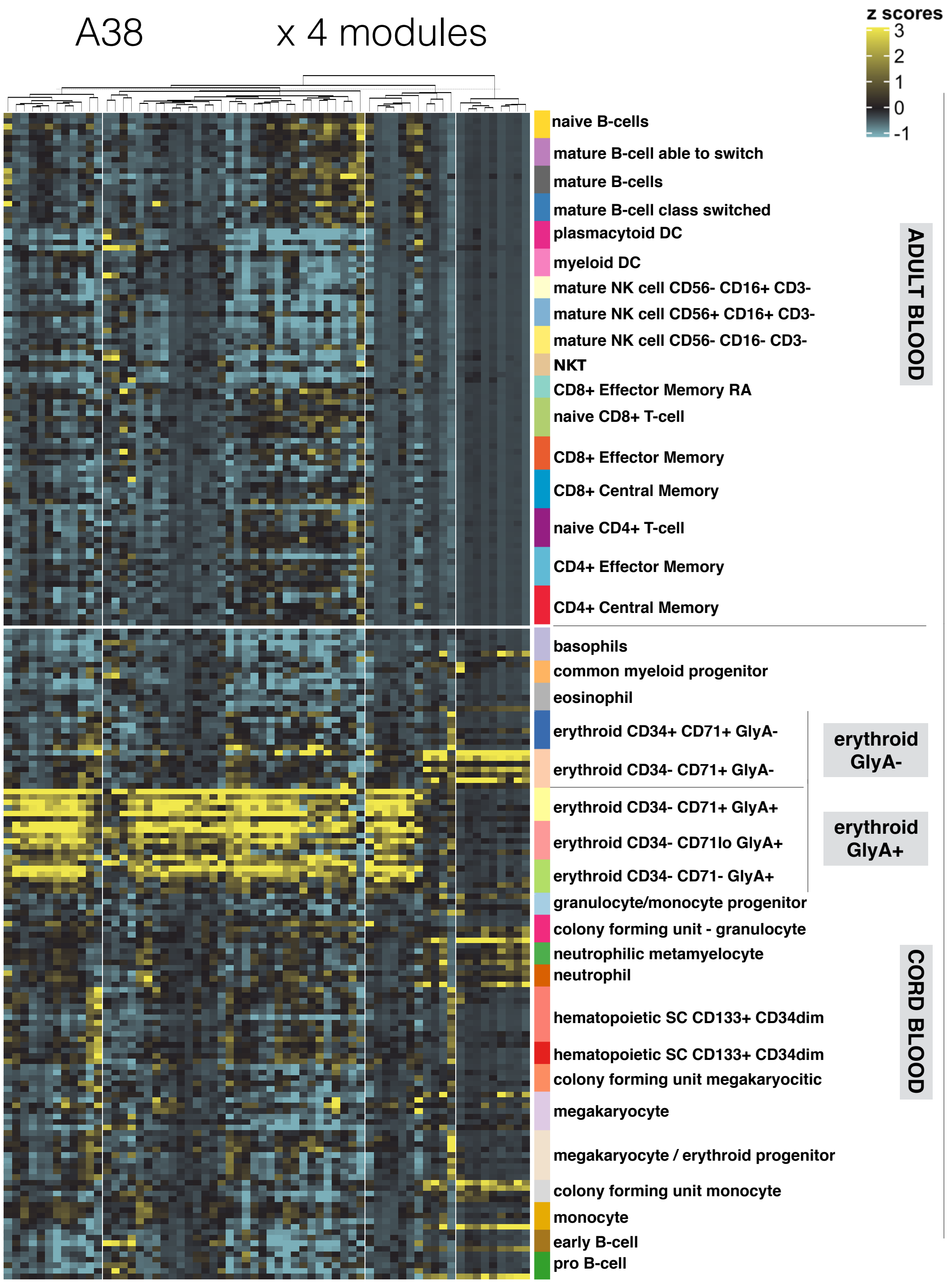

Supplementary Figure 7: The expression levels of A38 genes across the cell populations isolated from human peripheral blood and cord blood. The abundance levels of transcripts comprised in the 11 modules constituting aggregate A38 (columns) across blood cell populations (rows). The dataset is publicly available under GEO accession ID GSE24759 (24). The populations are separated based on whether they were isolated from adult venous blood (top) or from neonate cord blood (bottom). Distinct erythroid cell populations isolated on the basis of cell surface expression of CD34, CD71 and GlyA antigens are shown.
